# ZO-1 regulates the migration of mesenchymal stem cells in cooperation with α-catenin in response to breast tumor cells

**DOI:** 10.1038/s41420-023-01793-4

**Published:** 2024-01-11

**Authors:** Aran Park, Sanghyuk Choi, Jungbeom Do, Youngjae Kim, Kyung-Sup Kim, Eunjin Koh, Ki-Sook Park

**Affiliations:** 1 Graduate School of Biotechnology, Kyung Hee University, Yongin, 17104 Korea; 2 Department of Biomedical Science and Technology, Graduate School, Kyung Hee University, Seoul, 02447 Korea; 3 Department of Biochemistry and Molecular Biology, Institute of Genetic Science, Yonsei University College of Medicine, Seoul, 03722 Korea; 4 East-West Medical Research Institute, Kyung Hee University, Seoul, 02447 Korea

**Keywords:** Cancer microenvironment, Collective cell migration, Tight junctions

## Abstract

Mesenchymal stem cells are recruited from the bone marrow into breast tumors, contributing to the creation of a tumor microenvironment that fosters tropism for breast tumors. However, the intrinsic mechanisms underlying the recruitment of bone marrow-derived mesenchymal stem cells (MSCs) into the breast tumor microenvironment are still under investigation. Our discoveries identified zonula occludens-1 (ZO-1) as a specific intrinsic molecule that plays a vital role in mediating the collective migration of MSCs towards breast tumor cells and transforming growth factor beta (TGF-β), which is a crucial factor secreted by breast tumor cells. Upon migration in response to MDA-MB-231 cells and TGF-β, MSCs showed increased formation of adherens junction-like structures (AJs) expressing N-cadherin and α-catenin at their cell-cell contacts. ZO-1 was found to be recruited into the AJs at the cell-cell contacts between MSCs. Additionally, ZO-1 collaborated with α-catenin to regulate AJ formation, dependently on the SH3 and GUK domains of the ZO-1 protein. ZO-1 knockdown led to the impaired migration of MSCs in response to the stimuli and subsequent downregulation of AJs formation at the cell-cell contacts during MSCs migration. Overall, our study highlights the novel role of ZO-1 in guiding MSC migration towards breast tumor cells, suggesting its potential as a new strategy for controlling and re-engineering the breast tumor microenvironment.

## Introduction

Tumor cells orchestrate a tumor-supportive microenvironment by reprogramming resident non-transformed cells and recruiting non-transformed cells from distant tissues [1]. The interaction between tumor cells and the non-transformed cells composing the tumor microenvironment plays a critical role in coordinating the progression and metastasis of breast tumors [1, 2]. Investigating the mechanisms that drive the recruitment of non-transformed cells into the breast tumor microenvironment, as well as the mechanisms regulating the reprogramming of non-transformed cells within it, holds profound importance in advancing breast cancer therapeutic approaches focused on manipulating the microenvironment. Mesenchymal stem cells have been observed to be recruited from bone marrow into the breast tumor microenvironment and differentiate into stromal cells, such as cancer-associated fibroblasts exhibiting tumor-tropic characteristics [1, 3–6]. Hence, investigating the intrinsic mechanisms by which bone marrow-derived mesenchymal stem cells (MSCs) mediate their recruitment into the breast tumor microenvironment is crucial, even though these mechanisms have remained elusive.

Tight junctions are intercellular adhesion complexes that are primarily found in epithelial and endothelial cells and are essential for the barrier function of epithelial and endothelial cell layers [7]. However, mesenchymal cells do not typically form tight junctions. Tight junctions are composed of transmembrane proteins, like claudin and occludin, that interact with each other between neighboring cells. Inside the cells, these transmembrane proteins are linked to zonula occludens (ZO) proteins, which anchor them to the cytoskeleton, providing structural support and stability to the tight junctions [7]. ZO proteins includes ZO-1, ZO-2, and ZO-3, which are all scaffold proteins that play critical roles in the assembly and maintenance of tight junctions [7, 8]. ZO proteins are expressed also in the cells that lack the tight junctions and are involved in various cellular processes, such as cell polarity, proliferation, differentiation, and migration [7, 9–14]. While ZO-1 is primarily enriched at the tight junctions of epithelial and endothelial cells, it has also been found in various cancer cell lines and non-epithelial cell types such as astrocytes, schwann cells, and fibroblasts [15–17]. ZO-2 is also present in nonepithelial cells that lack tight junctions as well as in epithelial and endothelial cells [15, 18], but ZO-3 is primarily expressed in epithelial cells [19]. Hence, ZO-1 may play crucial roles in mesenchymal cells, which are neither epithelial nor endothelial, independent of its involvement in tight junctions. However, the specific functions of ZO-1 in mesenchymal cells have remained unclear.

Adherens junctions are specialized cell-cell junctions that are found in a variety of cell types, including epithelial, endothelial, and mesenchymal cells [20–23]. They are involved not only in the formation and maintenance of tissue architecture and cell-cell adhesion, but also in collective migration [20–23]. Cadherins are transmembrane proteins that mediate cell-cell adhesion at the adherens junctions and expressed in a tissue-specific manner; neural-cadherins (N-cadherin) is expressed primarily in the nervous system and mesenchymal cells [22]. At the adherens junctions, cadherins are linked to the actin cytoskeleton through a complex of cytoplasmic proteins, including p120, β-catenin, and α-catenin [20, 22]. α-Catenin interacts with ZO-1 in epithelial cells and non-epithelial mesenchymal cells through their direct interaction [24–27]. Therefore, the interaction between ZO-1 and α-catenin may be important for actin cytoskeleton remodeling associated with cell migration in mesenchymal cells, as well as for the functional coupling of adherens junctions and tight junctions in epithelial cells [24, 25].

Mesenchymal stem cells are recruited from bone marrow into breast tumors that secretes various chemotactic factors such as transforming growth factor beta (TGF-β), and contribute to form tumor microenvironment that enhance tumor progress and metastasis [3–5]. The intrinsic molecular mechanisms underlying the recruitment of MSCs to tumors are still not fully understood. However, there have been several proposed mechanisms. Our previous studies demonstrated that N-cadherin, the component of adherens junctions mediates the collective cell migration of MSCs into breast and prostate tumor cells [28, 29]. α-Catenin that regulates the formation and stability of the adherens junctions containing cadherins plays important roles in the collective cellular migration [30, 31]. Furthermore, ZO-1 plays an active role in cell migration by dynamically localizing to the leading edge of migrating cells and regulating the process [13, 32]. Additionally, ZO-1 associates with adherens junctions to regulate cell migration in various types of cells, including endothelial cells and cancer cells [12, 33]. This suggests that ZO-1 might also have a role in the formation of adherens junctions in MSCs and in mediating the migration of MSCs.

In the present study, we have newly identified the recruitment of ZO-1 into adherens junction-like structures (AJs) that express α-catenin and N-cadherin in MSCs. Furthermore, we demonstrated the pivotal role of ZO-1 in the formation of AJs at cell-cell contacts in MSCs, alongside the canonical component of adherens junctions, α-catenin. ZO-1 was observed to mediate the collective migration of MSCs toward breast tumor cells, as well as their migration in response to various factors, including TGF-β.

## Results

### ZO-1 and α-catenin mediate MSCs migration toward TGF-β

**Fig. 1:**
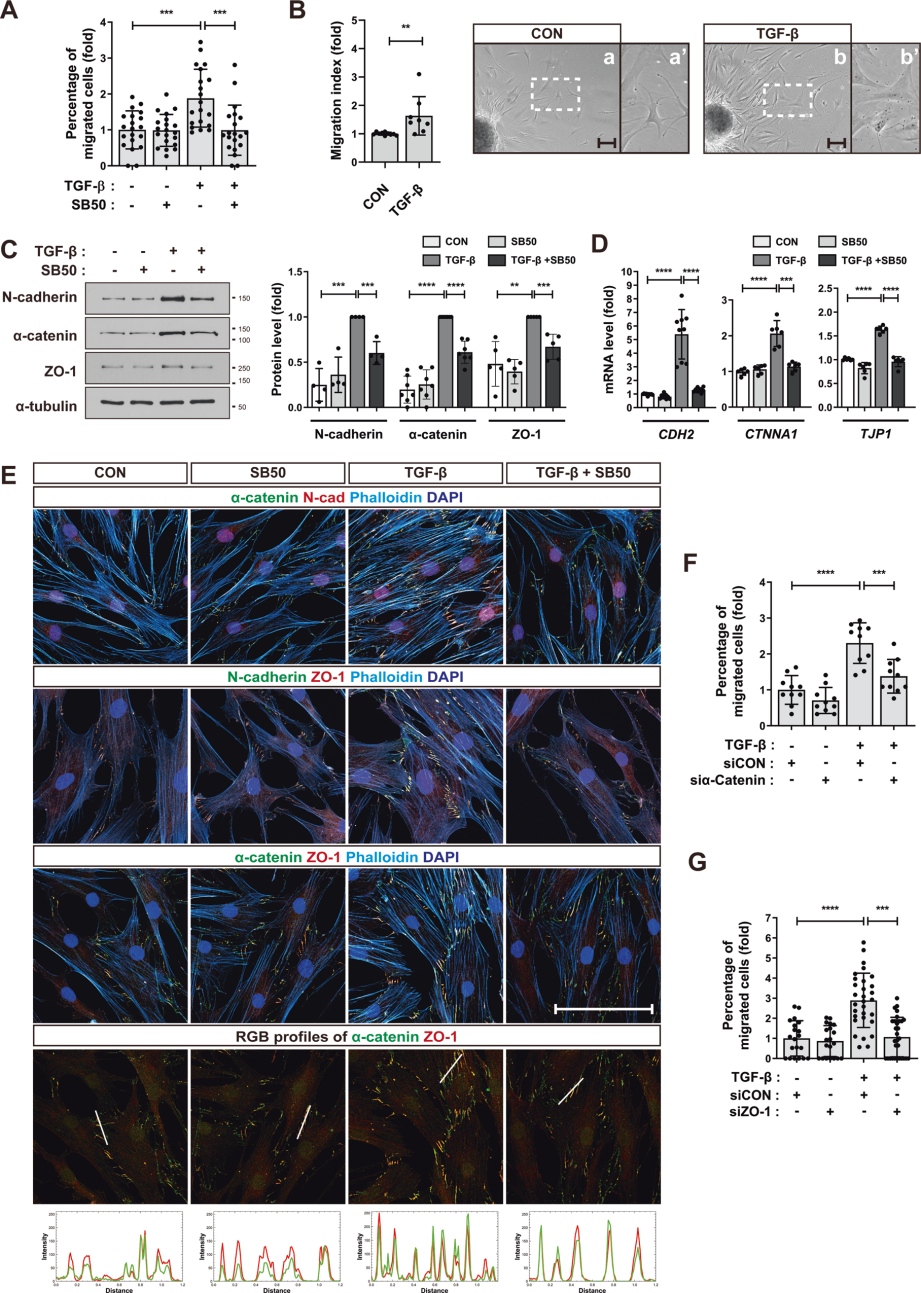
MSCs migrating toward TGF-β enhance the formation of ZO-1-associated AJs at the cell-cell contacts. **A** Transwell migration assay for quantifying MSCs migration in response to TGF-β. MSCs were treated with TGF-β (10 ng/ml) for 12 h. TGF-β type I receptor inhibitor, SB505124 (SB50, 500 nM) was pretreated for 30 min. The percentage of migrated cell was quantified. Representative images are shown in Supplementary Fig. S1A (*n* = 4 independent cultures). **B** Sphere migration assay for quantifying MSCs migration in response to TGF-β. MSCs spheres were treated with TGF-β (1 ng/ml) for 24 h. White dashed boxes in **a** and **b** are magnified in **a’** and **b’**, respectively (*n* = 2 independent cultures). **C** Western blot analysis of N-cadherin, α-catenin, and ZO-1. MSCs were pretreated with SB505124 (SB50, 500 nM) followed by the treatment of TGF-β (1 ng/ml) for 48 h. α-Tubulin was used as an internal control (*n* = 3 independent cultures). **D** Expression level of N-cadherin (*CDH2*), α-catenin (*CTNNA1*), and ZO-1 (*TJP1*) mRNA in MSCs pretreated with SB505124 (SB50, 500 nM) followed by the treatment of TGF-β (1 ng/ml) for 24 h (*n* = 3 independent cultures for N-cadherin, *n* = 2 independent cultures for α-catenin or ZO-1). **E** Immunostaining of N-cadherin, α-catenin, and ZO-1 in MSCs pretreated with SB505124 (SB50, 500 nM) followed by TGF-β (1 ng/ml) for 24 h. Actin and nuclei were stained with phalloidin (cyan blue) and DAPI (blue), respectively. RGB profiling was performed using ImageJ in the merged channels of ZO-1 and α-catenin. The analyzed regions are delineated by white lines (*n* = 3 independent cultures). **F**, **G** Transwell migration assay for quantifying the migration of α-catenin- (**F**) or ZO-1- (**G**) knockdown MSCs in response to TGF-β (10 ng/ml) for 12 h. Representative images are shown in Supplementary Fig. S1I, K (*n* = 2 independent cultures for (**F**) and *n* = 4 independent cultures for (**G**)). Scale bar, 100 μm. Results are presented as mean ± SD. *P* value measured by unpaired Student’s *t*-test; **P < 0.01, ***P < 0.001, ****P < 0.0001.

Bone marrow-derived mesenchymal stem cells (MSCs) migrated in response to TGF-β (Fig. 1A, B and Supplementary Fig. S1A) [28]. Our previous studies demonstrated that the expression of N-cadherin on MSCs is upregulated in a TGF-β-dependent manner [28, 34]. Furthermore, N-cadherin is found to localize at the adherens junction-like structures (AJs) at the cell-cell contacts between MSCs, playing a crucial role in regulating the collective migration of MSCs towards TGF-β [28, 34]. The expression of α-catenin, which links the adherens junction to actin cytoskeleton, increased in MSCs in response to TGF-β. SB505124, an inhibitor of TGF-β type I receptor impaired TGF-β-induced increase in α-catenin expression (Fig. 1C, D). In addition, α-catenin was colocalized with N-cadherin at AJs on the cell-cell contacts in TGF-β-treated MSCs (Fig. 1E). TGF-β upregulated the formation of AJs at cell-cell contacts as evidenced by the increase in length of α-catenin-positive structures at the cell-cell contacts in a TGF-β-dependent manner (Fig. 1E and Supplementary Fig. S1B, C). The width of the cell-cell contacts containing α-catenin-positive AJs also increased (Fig. 1E and Supplementary Fig. S1B, C). ZO-1 has been shown to play essential roles in crosslinking between adherens junctions and actin cytoskeleton in epithelial cells [24, 25]. MSCs exhibited the expression of ZO-1 and ZO-2 but lacked expression of ZO-3 (Supplementary Fig. S1D). The expression level of ZO-1 was higher than of ZO-2 and was comparable to that observed in endothelial cells (Supplementary Fig. S1D). In MSCs treated with TGF-β, there was an upregulation of ZO-1 expression, while ZO-2 expression showed a downregulation (Fig. 1C, D and Supplementary Fig. S1E). This highlights the distinct response of ZO-1 within MSCs. In addition, ZO-1 was concentrated at AJs on the cell-cell contacts of TGF-β-treated MSCs (Fig. 1E). Notably, ZO-1 was colocalized with N-cadherin, α-catenin, β-catenin, and p120 at AJs on MSCs (Fig. 1E and Supplementary Fig. S1F). The length of ZO-1-positive structures at the cell-cell contacts increased in response to TGF-β and the width of the cell-cell contacts containing ZO-1-positive AJs also increased (Supplementary Fig. S1G). The role of α-catenin and ZO-1 at AJs concerning the migration of MSCs towards TGF-β was investigated using Transwell migration assay. α-Catenin knockdown mediated through siRNA (Supplementary Fig. S1H) resulted in a decrease in the migration of MSCs towards TGF-β (Fig. 1F and Supplementary Fig. S1I). When ZO-1 expression was reduced through siRNA-mediated knockdown (Supplementary Fig. S1J), it led to a decrease in the migration of MSCs towards TGF-β (Fig. 1G and Supplementary Fig. S1K). Therefore, these results suggest that TGF‐β upregulated the formation of AJs at the cell-cell contacts of MSCs. In these structures, ZO-1, N-cadherin, and α-catenin were colocalized. ZO-1 and α-catenin in AJs mediated the migration of MSCs toward TGF‐β.

### ZO-1 is associated with AJs of MSCs

**Fig. 2:**
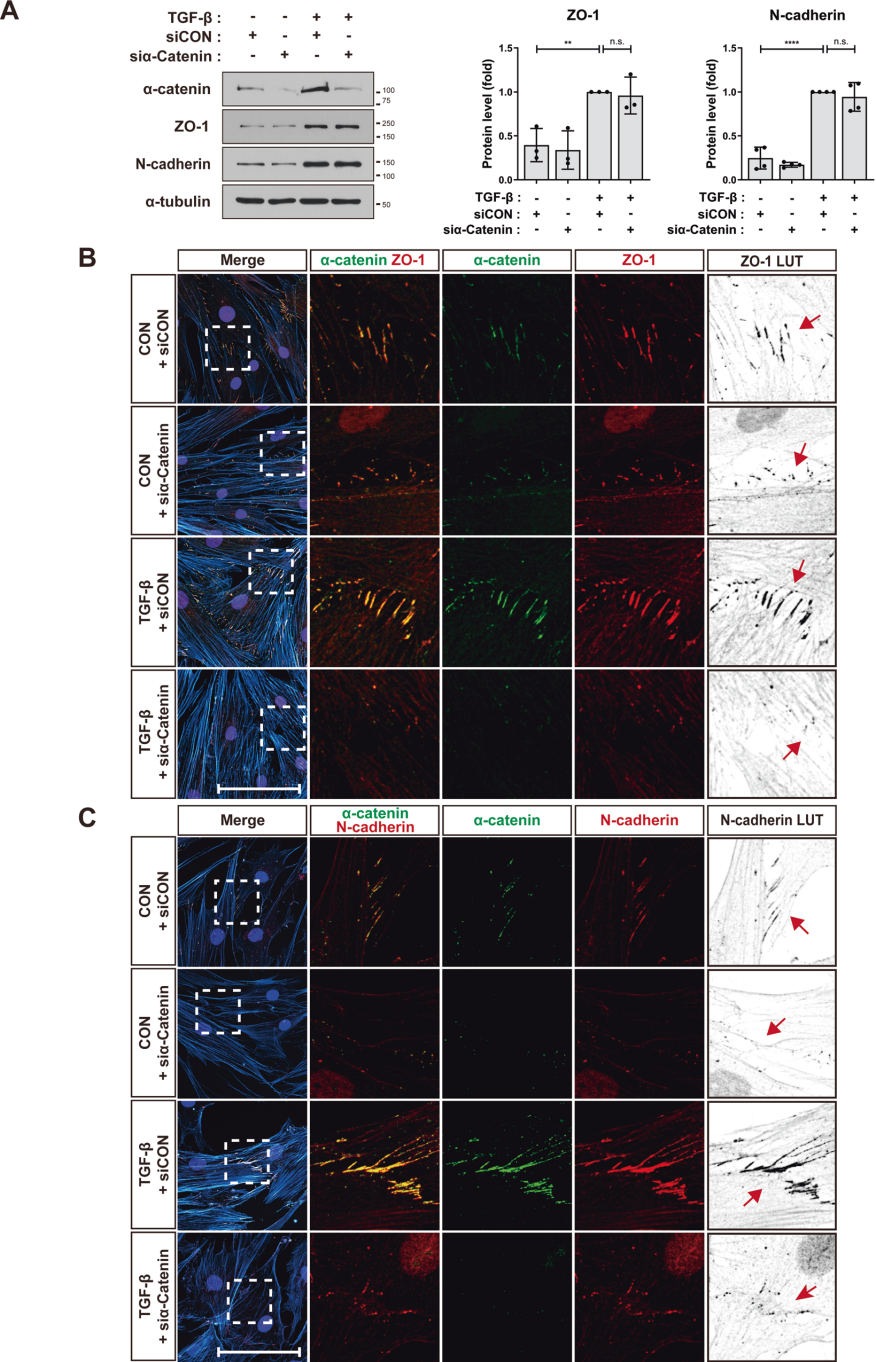
α-Catenin is required to recruit ZO-1 at the cell-cell contacts. **A** Western blot analysis of α-catenin, ZO-1, and N-cadherin in MSCs that were treated with TGF-β (1 ng/ml) for 48 h after the transfection of α-catenin siRNA (siα-Catenin) or the control siRNA (siCON). α-Tubulin was used as an internal control (*n* = 2 independent cultures). **B**, **C** Immunostaining of α-catenin and ZO-1 (**B**) or of α-catenin and N-cadherin (**C**) in α-catenin-knockdown MSCs treated TGF-β (1 ng/ml) for 24 h. Actin and nuclei were stained with phalloidin (cyan blue) and DAPI (blue), respectively. To enhance the visibility of ZO-1 (**B**) or N-cadherin (**C**), LUT (look up table) inverted images are presented. Red arrows indicate the cell-cell contacts in the regions magnified from white dashed boxes (*n* = 3 independent cultures). Scale bar, 100 μm. Results are presented as mean ± SD. *P* value measured by unpaired Student’s *t*-test; **P < 0.01, ****P < 0.0001, n.s. not significant.

**Fig. 3:**
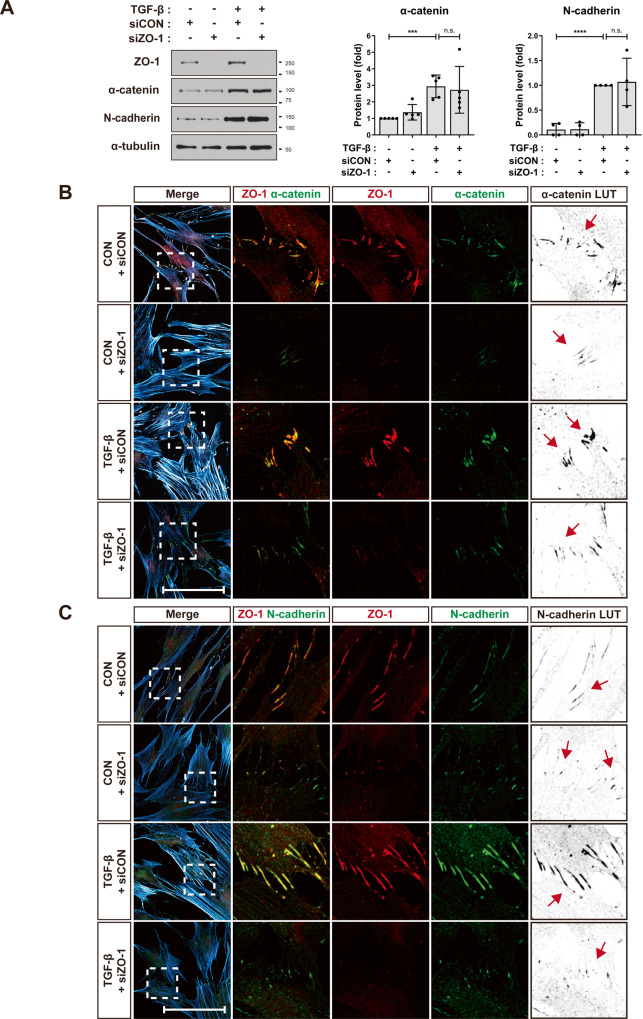
The knockdown of ZO-1 impairs the localization of α-catenin at AJs. **A** Western blot analysis of ZO-1, α-catenin, and N-cadherin in MSCs that were treated with TGF-β (1 ng/ml) for 48 h after the transfection of ZO-1 siRNA (siZO-1) or the control siRNA (siCON). α-Tubulin was used as an internal control (*n* = 2 independent cultures). **B**, **C** Immunostaining of ZO-1 and α-catenin (**B**) or ZO-1 and N-cadherin (**C**) in ZO-1-knockdown MSCs treated with TGF-β (1 ng/ml) for 24 h. Actin and nuclei were stained with phalloidin (cyan blue) and DAPI (blue), respectively. To enhance the visibility of α-catenin (**B**) or N-cadherin (**C**), LUT inverted images are presented. Red arrows indicate the cell-cell contact in the regions magnified from white dashed boxes. Scale bar, 100 μm. Results are presented as mean ± SD. *P* value measured by unpaired Student’s *t*-test; ***P < 0.001, ****P < 0.0001, n.s., not significant.

Based on the observed co-localization of ZO-1 with N-cadherin and α-catenin at AJs of MSCs, we investigated the potential mutual regulation of their localization at AJs. The knockdown of α-catenin did not alter TGF-β-induced increase in the expression of ZO-1 and N-cadherin in MSCs (Fig. 2A and Supplementary Fig. S2A). ZO-1 knockdown did not disrupt TGF-β-induced increase in the expression of α-catenin in MSCs, nor did it interfere with TGF-β-induced upregulation of N-cadherin expression (Fig. 3A and Supplementary Fig. S2B). Therefore, ZO-1 and α-catenin did not mutually regulate their expression in MSCs. Then, we investigated if ZO-1 and α-catenin reciprocally regulate their subcellular localization in MSCs. α-Catenin knockdown disrupted the TGF-β-induced concentration of ZO-1 and N-cadherin at AJs on the cell-cell contacts between MSCs (Fig. 2B, C and Supplementary Fig. S2C). ZO-1 knockdown impaired localization of α-catenin at AJs on the cell-cell contacts between MSCs (Fig. 3B and Supplementary Fig. S2D). Additionally, ZO-1 knockdown-induced disorganization of α-catenin localization corresponded to the mislocalization of N-cadherin at AJs in response to ZO-1 knockdown (Fig. 3C). However, the knockdown of N-cadherin, which was colocalized with ZO-1 and α-catenin, did not affect the expression or localization of ZO-1 and α-catenin at AJs between MSCs (Supplementary Fig. S3). In N-cadherin-knockdown MSCs, α-catenin was recruited to the cell-cell contacts (Supplementary Fig. S3C). Furthermore, ZO-1 was observed to localize at these sites as well (Supplementary Fig. S3D).

**Fig. 4:**
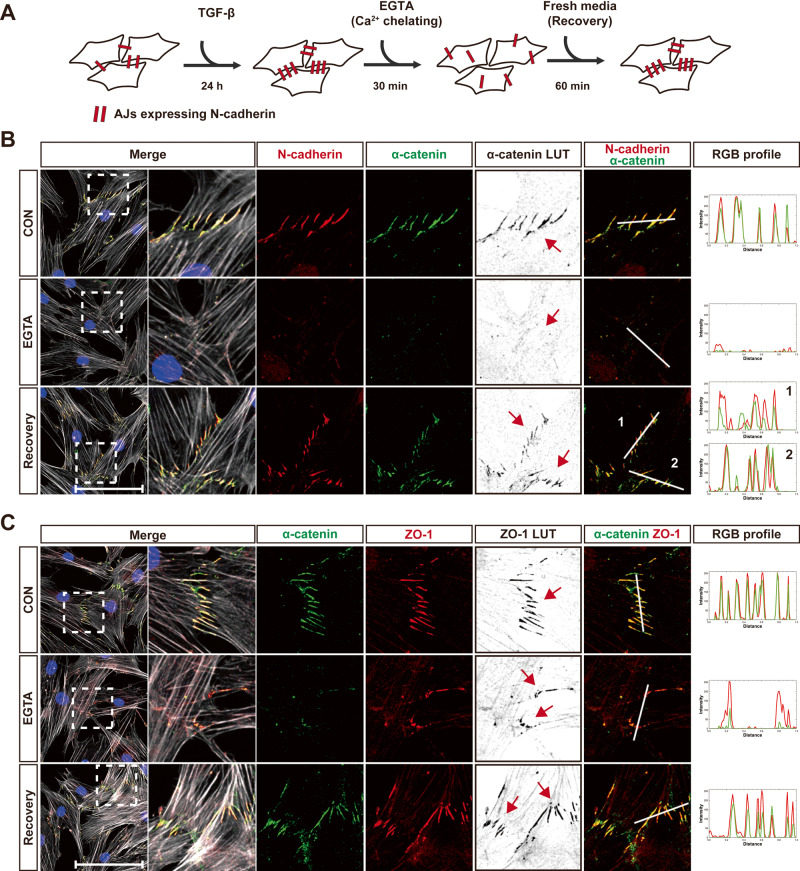
Inhibition of AJs formation impairs ZO-1 recruitment to the cell-cell contacts. **A** A schematic drawing of experimental procedure. **B**, **C** Immunostaining of N-cadherin and α-catenin (**B**) or α-catenin and ZO-1 (**C**) in MSCs. Actin and nuclei were stained with phalloidin (gray) and DAPI (blue), respectively. To enhance the visibility of α-catenin (**B**) or ZO-1 (**C**), LUT (look up table) inverted images are presented. Red arrows indicate the cell-cell contact in the regions magnified from white dashed boxes. RGB profiling was performed using ImageJ in the merged channels of N-cadherin and α-catenin (**B**) or α-catenin and ZO-1 (**C**). The analyzed regions are delineated by white lines, and white lines labeled with a number are corresponded to the RGB profiles from the right panel (*n* = 5 independent cultures for N-cadherin, *n* = 6 independent cultures for α-catenin, *n* = 3 independent cultures for ZO-1). Scale bar, 100 μm.

Then, after impairing the formation of AJs at the cell-cell contacts of MSCs treated with TGF-β, the localization of ZO-1 was investigated. The exogenous calcium chelator, ethylene glycol tetraacetic acid (EGTA) [35] distrupted AJs on the cell-cell contacts between MSCs treated with TGF-β (Fig. 4A). When AJs of MSCs were allowed to recover in normal fresh culture media, the impaired localization of α-catenin and N-cadherin was restored at AJs on the cell-cell contacts (Fig. 4B). Importantly, EGTA adversely affected the localization of ZO-1 at AJs as well as of N-cadherin and α-catenin (Fig. 4C). Upon restoration of AJs, ZO-1, along with α-catenin, was relocalized at AJs on the cell-cell contacts of MSCs (Fig. 4C). Consequently, these results suggest that ZO-1 is recruited to AJs formed at the cell-cell contacts of MSCs, along with cadherin and α-catenin, indicating that there is a close association between AJs formation and ZO-1 localization at cell-cell contacts in MSCs.

### ZO-1 regulates the formation of AJs on MSCs

Cadherin-based AJs at the cell-cell contacts have been shown to play essential roles in the migration of cancer cells [23, 36] and various mesenchymal-like cell types, including mesenchymal stem cells and neural crest cells [28, 29, 37]. The dynamic regulation of AJs is essential for governing cellular migration. Specifically, α-catenin regulates the formation of the initial contacts between neighboring cells and initiates AJs assembly [38]. Extensive evidence supports the pivotal role of ZO-1, a critical component of tight junctions, in the regulation of AJs composed predominantly of cadherin molecules and catenins [12]. The co-localization of ZO-1 and N-cadherin on melanoma cells was found to be essential for both invasion and adhesion processes [33]. ZO-1 has been shown to interact with α-catenin through their direct interaction [24–27]. ZO-1 may be functionally associated with AJs of MSCs and contributes to the regulation of AJs formation at the cell-cell contacts between MSCs through its mutual regulation with α-catenin. Therefore, our investigation focused on determining the regulatory role of ZO-1 in forming AJs at the cell-cell contacts of MSCs.

**Fig. 5:**
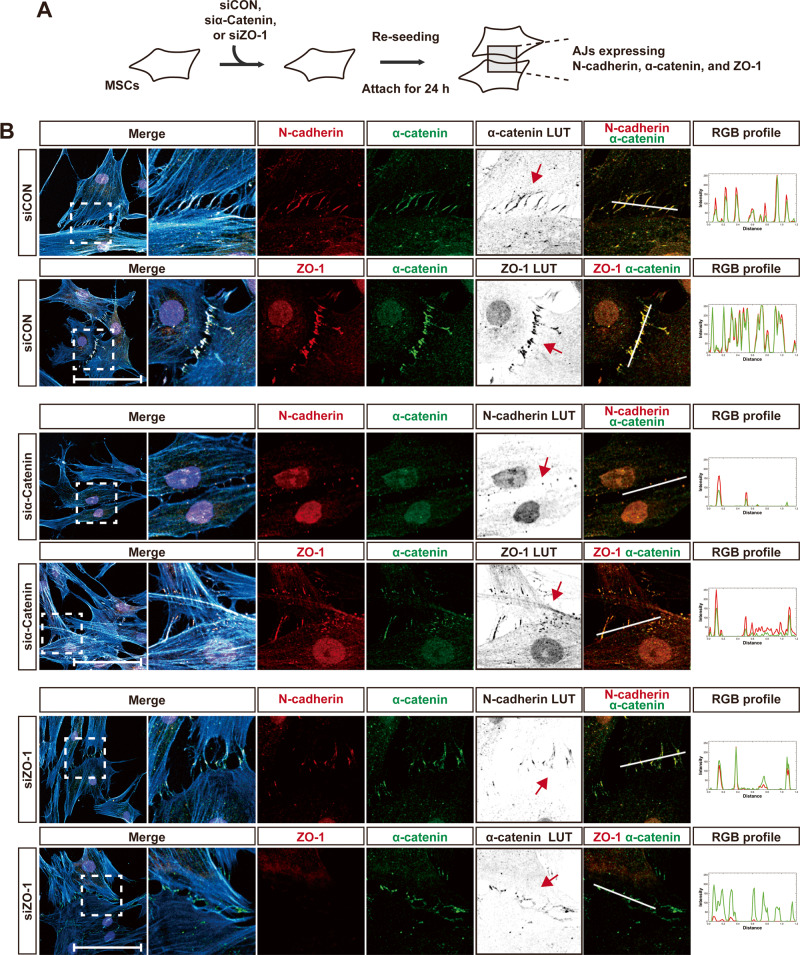
α-Catenin and ZO-1 are required for forming new AJs. **A** A schematic drawing of experimental procedure; siCON, the control siRNA; siα-Catenin, α-catenin siRNA; siZO-1, ZO-1 siRNA. **B** Immunostaining was performed to determine the subcellular localization of N-cadherin and α-catenin, as well as ZO-1 in MSCs that were transfected with siCON, siα-Catenin, or siZO-1. Actin and nuclei were stained with phalloidin (cyan blue) and DAPI (blue), respectively. To enhance the visibility of α-catenin, N-cadherin, or ZO-1, LUT (look up table) inverted images are presented. Red arrows indicate the cell-cell contact in the regions magnified from white dashed boxes. RGB profiling was performed using ImageJ in the merged channels of each identified in the figure. The analyzed regions are delineated by white lines (*n* = 2 independent cultures). Scale bar, 100 μm.

ZO-1-knockdown or α-catenin-knockdown MSCs were seeded to allow the formation of new AJs at the cell-cell contacts (Fig. 5A). The control MSCs established the cell-cell contacts and AJs were successfully formed at the cell-cell contacts. At the cell-cell contacts of the control MSCs, AJs expressing N-cadherin were observed (Fig. 5B). The α-catenin-positive signal completely overlapped with the N-cadherin-positive signal at AJs and the ZO-1-positive signal overlapped with the α-catenin-positive signal (Fig. 5B). However, in α-catenin-knockdown MSCs, the formation of AJs at the cell-cell contacts decreased (Fig. 5B). The number of AJs expressing N-cadherin was lower than in the control. Moreover, ZO-1 recruitment into AJs was also downregulated in α-catenin-knockdown MSCs, compared to the control (Fig. 5B). Similarly, the formation of AJs at the cell-cell contacts was reduced in ZO-1-knockdown MSCs in contrast to the control (Fig. 5B). The frequency of AJs expressing α-catenin and N-cadherin was lower in ZO-1-knockdown MSCs than in the control MSCs. Therefore, the results suggest that ZO-1 plays a role in regulating the formation of α-catenin-expressing AJs at the cell-cell contacts of MSCs.

### ZO-1 is recruited into AJs on MSCs via SH3 and GUK domains

ZO-1 consists of several domains, including three PDZ domains, a SH3 domain, a GUK domain, and actin binding region (Supplementary Fig. S4A) [39]. ZO-1 interacts with claudin via its PDZ domain [39]. Additionally, ZO-1 directly interacts with occludin or α-catenin through its region that includes SH3 domain and GUK domain [40]. Occludin expression level was lower in MSCs than in HUVECs, endothelial cells (Supplementary Fig. S4B). This suggests that α-catenin may interact with ZO-1 in MSCs in the absence of occludin competition. Overall, these imply that the SH3 and GUK domains of ZO-1 might play a role in recruiting ZO-1 into adherens junctions (AJs) including α-catenin in MSCs. This contribution could be vital for ZO-1-dependent regulation of AJs formation at the cell-cell contacts of MSCs.

**Fig. 6:**
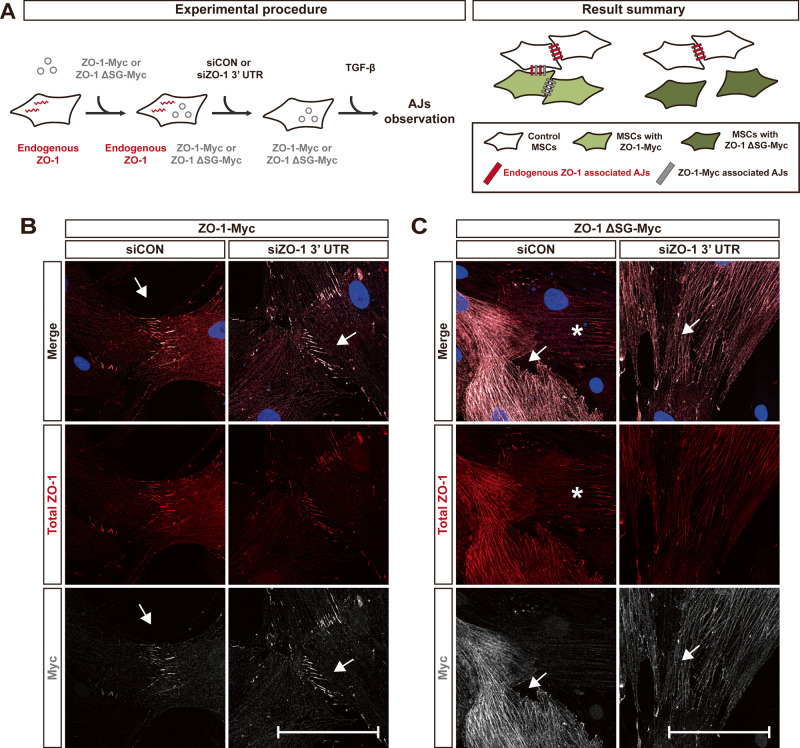
The SH3 and GUK domains in ZO-1 play a crucial role in recruiting ZO-1 to AJs at the cell-cell contact. **A** A schematic drawing of experimental procedure and result summary. Immunostaining of ZO-1 (red), Myc (gray), and DAPI (blue) in MSCs. MSCs infected with full-length ZO-1 tagged with Myc (ZO-1-Myc) (**B**) or a mutant ZO-1 lacking SH3 and GUK domains tagged with Myc (ZO-1 ΔSG-Myc) (**C**) were transfected with either the control siRNA (siCON) or siRNA targeting ZO-1 3′ UTR (siZO-1 3′ UTR). White arrows indicate the cell-cell contacts and white asterisks (*) indicate MSCs expressing endogenous ZO-1, but not expressing the mutant ZO-1. Scale bar, 100 μm.

We investigated the requirement of the region of ZO-1, which includes the SH3 domain and GUK domain, for forming AJs at the cell-cell contacts MSCs, where α-catenin is concentrated in response to TGF-β as shown in Fig. 1. Firstly, the endogenous expression of ZO-1 was knocked down in MSCs using siRNA specific to the ZO-1 3′-UTR (Fig. 6A and Supplementary Fig. S4C, D). Subsequently, full-length ZO-1 tagged with Myc or a mutant form of ZO-1 tagged with Myc (truncated in the region containing the SH3 domain and GUK domain; Supplementary Fig. S4A) was introduced in MSCs. (Fig. 6B, C and Supplementary Fig. S4D). The full-length ZO-1, tagged with Myc, was observed to localize at AJs on the cell-cell contacts of MSCs (Fig. 6B). However, the mutant form of ZO-1, lacking the region including the SH3 domain and GUK domain, did not exhibit localization at the contacts of MSCs (Fig. 6C). The endogenous ZO-1 was recruited into the cell-cell contacts between the control MSCs, which expressed the endogenous ZO-1. However, this recruitment was not observed when one of two contacting MSCs expressed the mutant form of ZO-1 (Fig. 6C; white asterisks indicate MSCs that expressed only the endogenous ZO-1). Thus, these results suggest that the region of ZO-1 encompassing the SH3 and GUK domains is crucial for ZO-1 recruitment into AJs on the cell-cell contacts of MSCs.

### ZO-1 is required for the collective migration of MSCs toward breast tumor cells

**Fig. 7:**
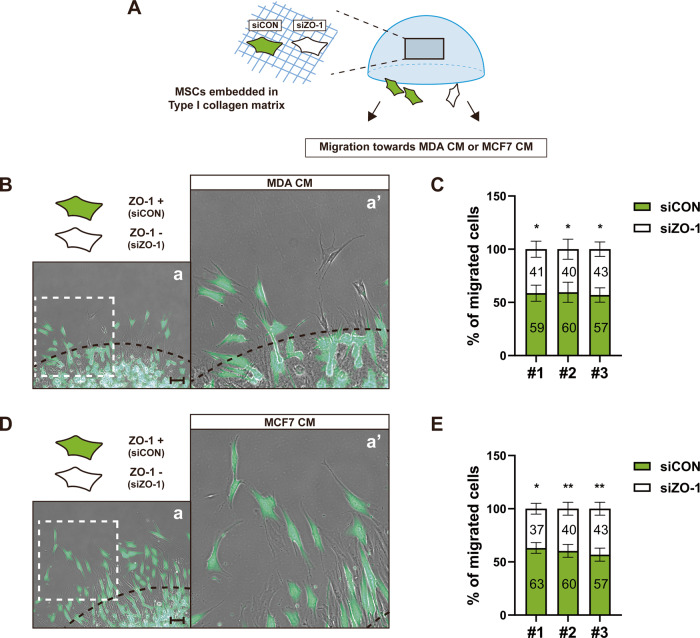
ZO-1 mediates the 3D migration of MSCs in response to the conditioned media of breast tumor cells. **A** A schematic drawing of experimental procedure and result summary. MSCs transfected with the control siRNA (siCON) were labeled with CellTracker Green CMFDA (10 μM) and mixed with MSCs transfected with ZO-1 siRNA (siZO-1) within a collagen matrix. Subsequently, the collagen gel containing the mixture of the cells were treated with MDA CM or MCF7 CM and incubated for 48 h. **B**, **D** Representative images of MSCs that migrated out from the collagen gel in response to MDA CM (**B**) or MCF7 CM (**D**). Black dashed lines indicate the margin of collagen gel. White dashed boxes in (**a**) are magnified in (**a’**). The percentage of MSCs transfected with siCON or siZO-1 that migrated out from the collagen gel in response to MDA CM (**C**) or MCF7 CM (**E**) (*n* = 3 independent cultures for **C** and *n* = 3 independent cultures for **E**). Scale bar, 100 μm. Results are presented as mean ± SD. *P* value measured by unpaired Student’s *t*-test; *P < 0.05, **P < 0.01.

**Fig. 8:**
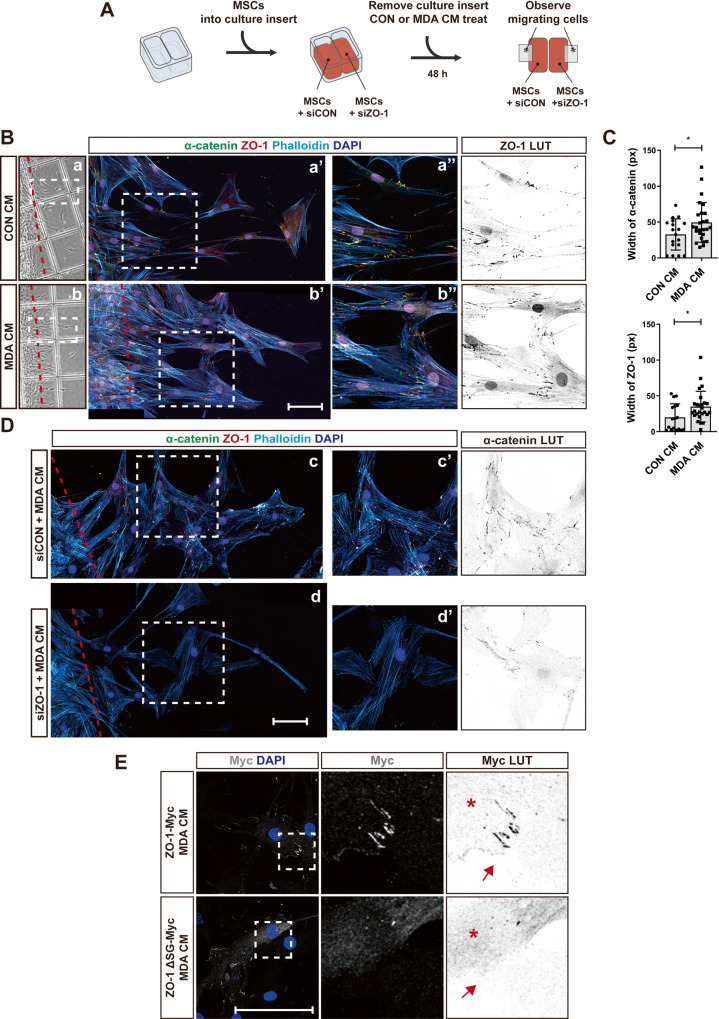
ZO-1 is recruited into AJs at the cell-cell contacts of MSCs collectively migrating towards breast tumor cells. **A** A schematic drawing of experimental procedure for Two-well insert migration assay. MSCs transfected with the control siRNA (siCON) or ZO-1 siRNA (siZO-1) were seeded into each well. After removing the insert, MDA CM was applied and incubated for 48 h. Immunostaining of α-catenin, ZO-1, phalloidin, and DAPI in MSCs (**B**) and the quantification of the width of the cell-cell contacts containing α-catenin or ZO-1 positive structures (**C**; more than 18 cell-cell contacts were analyzed). **D** Immunostaining of α-catenin, ZO-1, phalloidin, and DAPI in MSC transfected with siCON or siZO-1. The red dashed lines indicate the margin of the insert. White dashed boxes in **a,** **b,** **c**, and **d** are magnified in **a’,** **b’,** **c’**, and **d’**, respectively. White dashed boxes in **a’,** **b’,** **c’**, and **d’** are magnified in **a”,** **b”,** **c”**, and **d”**, respectively. To enhance visibility of ZO-1 and α-catenin, LUT inverted images are presented. **E** Immunostaining of overexpressed full-length ZO-1 (ZO-1-Myc) or the mutant ZO-1 (ZO-1 ΔSG-Myc) in MSCs. Myc (gray) tag and DAPI (blue) were stained and Myc visualization was enhanced using LUT inverted images. Red arrows indicate the cell-cell contacts in the regions magnified from white dashed boxes and cells expressing exogenous ZO-1 are marked with asterisk (*). Scale bar, 100 μm. Results are presented as mean ± SD. *P* value measured by unpaired Student’s *t*-test; *P < 0.05.

MSCs were able to migrate into various stimuli including TGF-β. The migration of MSCs toward stromal cell-derived factor-1 (SDF-1) or 10% fetal bovine serum (FBS) was impaired with ZO-1 knockdown (Supplementary Fig. S5A). MSCs migrated towards in vitro conditions mimicking the tumor microenvironment in a ZO-1-dependent manner. The conditioned medium (CM) derived from breast tumor cells including MDA-MB-231 and MCF7, prostate tumor cells, or glioblastoma cells simulated the migration of MSCs. The migration of MSCs towards the conditioned media was inhibited when ZO-1 was knocked down in MSCs (Supplementary Fig. S5B–D). In our previous study, we demonstrated that MSCs collectively migrate in response to breast tumor cells [28]. Furthermore, during migration, MSCs maintained N-cadherin-positive AJs at the cell-cell contacts [28]. ZO-1 was recruited into AJs and regulated AJ formation at the cell-cell contacts of MSCs that migrated in response to TGF-β (Figs. 1–6). Therefore, we investigated the potential role of ZO-1 in mediating the collective migration of MSCs towards breast tumor cells. A three-dimensional (3D) cell migration assay was performed using a mixture of the control MSCs expressing ZO-1 and ZO-1-knockdown MSCs (Fig. 7A). MSCs lacking ZO-1 exhibited reduced migration when exposed to the conditioned medium derived from MDA-MB-231 (MDA CM) or from MCF7 (MCF7 CM), compared to the control cells with intact ZO-1 expression (Fig. 7B–E). Then, AJs at the cell-cell contacts between MSCs migrating in response to MDA CM was investigated using two-well culture insert (Fig. 8A). MSCs were cultured in both sides of the culture inserts and the culture insert was removed to facilitate cell migration. More MSCs collectively migrated in response to MDA CM, compared to the control conditioned media (CON CM). During their collective migration, MSCs maintained α-catenin-positive AJs at the cell-cell contacts (Fig. 8B). Additionally, ZO-1 was observed to colocalize on AJs with α-catenin at the cell-cell contacts between collectively migrating MSCs (Fig. 8B). In response to MDA CM, the width of the cell-cell contacts containing α-catenin-positive or ZO-1-positive AJs increased on collectively migrating MSCs, compared to CON CM (Fig. 8C). Furthermore, ZO-1, which was crucial for the collective migration of MSCs in response to MDA CM, was also essential for preserving AJs at the cell-cell contacts during the collective migration process. In the ZO-1-knockdown MSCs, the presence of α-catenin-positive AJs was lacking at the cell-cell contacts of MSCs migrating toward MDA CM (Fig. 8D). In addition, ZO-1 mutant that lacked the ability for forming AJs at the cell-cell contacts was not recruited on the cell-cell contacts between the control MSCs and MSCs expressing ZO-1 mutant under MDA CM condition (Fig. 8E). These results suggest that ZO-1 is recruited into AJs at the cell-cell junctions and plays an essential role in preserving AJs on the cell-cell contacts during the collective migration of MSCs.

## Discussion

In this study, we investigated the potential role of ZO-1 in mediating MSCs migration towards TGF-β and breast tumor cells. Our findings revealed that ZO-1 was recruited into AJs and played a crucial role in regulating the formation of AJs at the cell-cell contacts of migrating MSCs, in response to TGF-β stimulation and to in vitro conditions mimicking breast tumors. These results shed light on the mechanistic aspects of MSC migration toward the tumor microenvironment and highlight the importance of ZO-1 in facilitating this process.

MSCs possess the multilineage differentiation potential [41, 42]. MSCs are recruited into injury tissues to facilitate the regeneration of the injury [43–46]. MSCs also infiltrate the tumor microenvironment, where they interact with both tumor cells and non-transformed cells, thereby promoting the proliferation and metastasis of the tumor cells [3, 47–49]. Therefore, MSCs require recruitment into tumors and subsequent local migration into neighboring cells within the tumor microenvironment to facilitate the interactions. ZO-1 has been shown to mediate the cellular migration of various cell types [12–14, 32, 33, 50–52]. The regulation of VE-cadherin-dependent junctions is crucial for the migration and rearrangement of endothelial cells, and ZO-1 plays a role in fine-tuning these VE-cadherin-dependent junctions [12, 52]. AJs expressing N-cadherin on the cell-cell contacts between MSCs have been shown to be necessary for the collective migration of MSCs towards the breast tumor cells and prostate tumor cells [28, 29], but it has been entirely unknown whether ZO-1 plays a role in regulating the migration of MSCs and the formation of AJs on these cells.

The current study demonstrated the cooperation between ZO-1 and α-catenin was required for the collective migration of MSCs. α-Catenin, a crucial and fundamental element involved in cadherin-dependent cell-cell adhesions, has the capacity for indirect interaction with cadherins [22, 26] as well as direct interaction with ZO-1 [24, 25, 40]. Therefore, it is possible that α-catenin mediates the interaction between cadherins and ZO-1 in MSCs, facilitating the migration of MSCs expressing both cadherins and ZO-1. MSCs exhibited reciprocal regulation-dependent localization of ZO-1 and α-catenin on AJs expressing N-cadherin at the cell-cell contacts. Furthermore, their expression increased in response to stimuli that induced MSCs migration. MSCs lacking ZO-1 or α-catenin exhibited the impaired migration capacity. Moreover, the ZO-1 mutant lacking the domains that may be necessary for interaction with α-catenin was unable to be recruited into AJs at the cell-cell contacts between MSCs. These results suggest that ZO-1 regulates the migration of MSCs towards breast tumor cells by modulating the formation of AJs at the cell-cell contacts on the migrating MSCs through its interaction with α-catenin. ZO-1 and α-catenin were colocalized at the cell-cell contacts in N-cadherin-knockdown MSCs. N-cadherin has been identified to be expressed on AJs at the cell-cell contacts of MSCs and is necessary for the collective migration of MSCs toward TGF-β and tumor cells [28, 29, 34]. In addition to N-cadherin, MSCs also expressed another cadherin, OB-cadherin, at the cell-cell contacts [53]. OB-cadherin may play a role in maintaining AJs in N-cadherin-knockdown MSCs. OB-cadherin has the potential to play a role in the formation of AJs alongside ZO-1 and α-catenin within N-cadherin-knockdown MSCs.

Following the knockdown of ZO-1 in MSCs, the expression of ZO-2 and ZO-3 remained unchanged, suggesting that ZO-1 might possess a distinct role in mediating MSC migration, independent of compensation from other ZO isoforms. However, it is not yet clear whether ZO-1 serves as an intrinsic factor in regulating the cellular migration of MSCs without causing any changes in their tumor tropic characteristics. Further studies are needed to investigate whether ZO-1 knockdown influences the expression of genes related to angiogenesis, inflammation cytokines, or stemness. Such research would provide valuable information about roles of ZO-1 in MSCs behavior within the context of tumor biology.

The current study bears significant implications for the advancement of technologies focused on controlling the breast tumor microenvironment. The observed involvement of ZO-1 in facilitating MSC migration towards breast tumor cells opens a promising avenue for the progress of anti-cancer treatments tailored for breast tumor. By utilizing the insights garnered from this study, novel strategies can be formulated to precisely regulate and manipulate the tumor microenvironment, thus enhancing the efficacy of therapeutic interventions. Recently, MSCs are being explored as carriers of anti-cancer therapeutic factors, primarily because of their tumor-homing capacity. Fine-tuning and facilitating MSCs recruitment towards tumors are crucial aspects of this development. ZO-1, which plays a role in mediating MSCs migration towards breast tumor cells, could potentially serve as a new target to enhance MSCs migration towards breast tumors.

## Materials and methods

### Cell culture

Human bone marrow-derived mesenchymal stem cells (MSCs) were obtained from Lonza (Basel, Switzerland) and were maintained with Mesenchymal Stem Cell Growth Medium (Lonza). The cells between passages 4 and 7 were used for all experiments. MDA-MB-231, MCF7, U87, PC3, BT474, and HEK293T cells were obtained from the American Type Culture Collection (ATCC, Manassas, VA, USA). T47D, LNCaP, SKBR3, and HEK293 cells were purchased from Korean Cell Line Bank (KCLB, Seoul, Republic of Kore). MDA-MB-231, BT474, SKBR3, and HEK293T cells were cultured in Dulbecco’s Modified Eagle medium/high glucose (DMEM/HG; Hyclone, Logan, UT, USA), supplemented with 10% heat-inactivated fetal bovine serum (FBS; Thermo Fisher Scientific, Waltham, MA, USA) and 100 U/ml penicillin/streptomycin (P/S; Thermo Fisher Scientific). PC3 cells were cultured in DMEM/HG supplemented with 10% FBS, 2 mM L-glutamine (Thermo Fisher Scientific), and 100 U/ml P/S. MCF7 cells were cultured in DMEM/F-12 (1:1) (Thermo Fisher Scientific) supplemented with 10% FBS, 2 mM L-glutamine (Thermo Fisher Scientific), and 100 U/ml P/S. LNCaP cells were maintained with Roswell Park Memorial Institute 1640 (RPMI1640, Thermo Fisher Scientific) supplemented with 10% FBS and 100 U/ml P/S. T47D cells were maintained with RPMI1640 supplemented with 10% FBS and 100 U/ml P/S. U87 and HEK293 cells were maintained with DMEM/low glucose (DMEM/LG) supplemented with 10% FBS, 2 mM L-glutamine, and 100 U/ml P/S. Human Umbilical Vein Endothelial Cells (HUVECs; Lonza) and human bone marrow-derived hematopoietic progenitors expressing CD34 (CD34+; STEMCELL Technologies, Vancouver, Canada) were thawed from cryovial to purify total RNA. All the cells for culture were maintained at 37 °C in a humidified incubator containing 5% CO_2_.

### Reagents

Recombinant human TGF-β (1 ng/μl or 10 ng/μl, R&D systems, Minneapolis, MN, USA) and recombinant mouse CXCL12/SDF-1α protein (50 ng/μl, R&D systems) were used on MSCs. SB505124 (500 nM, Sigma-Aldrich), an inhibitor of TGF-β type I receptor was pretreated for 30 min prior to TGF-β treatment to inhibit TGF-β signaling. As a calcium chelator, ethylene glycol-bis(2-aminoethylether)-N,N,N′,N′-tetraacetic acid (EGTA, 4 mM, Sigma-Aldrich) was used. MSCs were incubated with 10 μM of CellTracker™ Green CMFDA Dye (Thermo Fisher Scientific) in a serum-free condition for 30 min to visualize their migration. All the reagents were reconstituted and used according to the manufacturer’s instructions.

### Conditioned media preparation

Cancer cell lines were cultured until reaching confluence. Subsequently, the media for each cell line were replaced with their respective fresh culture media, containing reduced FBS compared to the complete culture media. Conditioned media for the migration assay were prepared by incubating MDA-MB-231, MCF7, U87, PC3, or LNCaP cells under serum-free conditions for 3 days. BT474, T47D, or SKBR3 cells were cultured with 2% FBS-containing media for 2 days. Conditioned media for the immunostaining of ectopic ZO-1 expression were prepared by incubating MDA-MB-231 cells with 2% FBS containing culture media for 2 days. To prepare conditioned media for the control, FBS-deprived or FBS-reduced media specific to each cell line were incubated for the same period under cell-free condition. The conditioned media were filtered through a 0.2 μm filter (Corning, Corning, NY, USA), aliquoted, and stored at −80 °C until use.

### Quantitative real-time polymerase chain reaction (qRT-PCR)

Total RNA was isolated with TRIzol reagent (Thermo Fisher Scientific), and cDNA was synthesized using SuperScript III Reverse Transcriptase (Thermo Fisher Scientific), according to the manufacturer’s protocols. Quantitative real-time polymerase chain reaction (qRT-PCR) was performed using the SYBR Green reagent (Thermo Fisher Scientific). The human ribosomal protein S9 gene (*RPS9*) was used as an endogenous control. The sequences of primers were as follows: *RPS9* (sense): 5′-CTGACGCTTGATGAGAAGGAC-3′, *RPS9* (antisense): 5′-CAGCTTCATCTTGCCCTCAT-3′; *CDH2* (sense): 5′-CATCCCTCCAATCAACTTGC-3′, *CDH2* (antisense): 5′-ATGTGCCCTCAAATGAAACC-3′; *CTNNA1* (sense): 5′-CAGCTAGCCGCAGAAATGAC-3′, *CTNNA1* (antisense): 5′- GAGGCTCCAACAGTCTCTCAA-3′; *TJP1* (sense): 5′-GTGTTGTGGATACCTTGT-3′, *TJP1* (antisense): 5′-GATGATGCCTCGTTCTAC-3′; *TJP2* (sense): 5′-AGCAGAGCGAACGAAGAGTA-3′, *TJP2* (antisense): 5′-TCTCCTTCGTGAAGGTTGCC-3′; *TJP3* (sense): 5′-CGAGAAGCCAGTTTCAAGCG-3′, *TJP3* (antisense): 5′-ATAGTTGAGGCGCTCGATGG-3′; *OCLN* (sense): 5′-TCAGGGAATATCCACCTATCACTTCAG-3′, *OCLN* (antisense): 5′-CATCAGCAGCAGCCATGTACTCTTCAC-3′.

### Transfection of cells with small interfering RNAs (siRNA)

MSCs were transfected with siRNA using Lipofectamine RNAiMAX (Thermo Fisher Scientific) following the manufacturer’s instructions. Trilencer-27 Universal scrambled negative control siRNA (control siRNA; ORIGENE, Rockville, MD, USA) was used as a negative control. siRNA of α-catenin, ZO-1, and N-cadherin were purchased from Thermo Fisher Scientific. ZO-1 siRNA targeting 3′ UTR was synthesized by Genolution (Seoul, Republic of Korea). The sequences were as follows: *TJP1* 3′ UTR siRNA (sense: 5′-AAGAUACAGUUACUCAGAAUU-3′; antisense: 5′-UUCUGAGUAACUGUAUCUUUU-3′).

### Western blotting

To evaluate the TGF-β-induced protein expression in MSCs, SB505124 (500 nM) was treated prior to the TGF-β treatment for 48 h. For analyzing reciprocal regulation between α-catenin or ZO-1 on their protein expression, the control siRNA, α-catenin siRNA, or ZO-1 siRNA was transfected into MSCs for 48 h and serum starved overnight prior to TGF-β treatment for 48 h. MSCs were washed with ice-cold Phosphate Buffered Saline (PBS; pH 7.0) with Ca^2+^ and Mg^2+^ and lysed with 2**×** SDS buffer (100 mM Tris-HCl, pH 6.8, 20% (v/v) glycerol, 2% (v/v) sodium dodecyl sulfate (SDS), 0.001% (w/v) bromophenol blue, and 10% (v/v) β-mercaptoethanol) at 25 °C for 5 min. Cell lysates were collected by scrapping and denaturated at 95 °C for 5 min. Proteins were separated on SDS-polyacrylamide gel and transferred to a nitrocellulose membrane (Bio-Rad, Hercules, CA, USA). Immunoblotting was performed by the following primary antibodies: anti-ZO-1 (Thermo Fisher Scientific, Waltham, MA, USA), anti-α-catenin (BD Biosciences, Franklin Lakes, NJ, USA), anti-N-cadherin (Thermo Fisher Scientific), and α-tubulin (Sigma-Aldrich). Densitometry of the bands was analyzed using the ImageJ software (NIH, Bethesda, MD, USA).

### Immunostaining and confocal microscopy analysis

MSCs were seeded onto Type I collagen (190 μg/ml; Nitta Gelatin NA Inc., Morrisville, NC, USA) coated cover glasses. The cells were washed with ice-cold PBS (pH 7.0) containing Ca^2+^ and Mg^2+^ and fixed with 4% formaldehyde (Sigma-Aldrich) for 10 min on ice. Permeabilization was conducted with 0.2% Triton X-100 (Sigma-Aldrich) in PBS, and 5% non-fat milk in 0.1% Triton X-100 in PBS was used for the blocking. The cells were incubated for 90 min at 25 °C with primary antibodies against ZO-1 (Thermo Fisher Scientific), α-catenin (BD Biosciences), N-cadherin (Thermo Fisher Scientific), N-cadherin (Takara, Shiga, Japan), or Myc-tag (Cell Signaling Technology). Nuclei were stained using 4,6-diamidino-2-phenylindole (DAPI; Thermo Fisher Scientific) for 10 min, and actin was detected using Alexa-Fluor-633-tagged phalloidin (Thermo Fisher Scientific). The covers were mounted on glass slides with Fluoromount-G (SouthernBiotech, Birmingham, AL, USA). Image acquisition was performed by Zeiss LSM 700 confocal microscope (Carl Zeiss, Oberkochen, Germany) and processed by Zeiss Zen (Blue edition) software. If necessary, consecutive stitch sequences were processed into a single image using Fiji 2.14 (NIH, Bethesda, MD, USA).

### Junctional reassembly assay

To observe the formation of new junctions dependent on α-catenin or ZO-1, MSCs were transfected with the control siRNA, α-catenin siRNA, or ZO-1 siRNA and incubated for 72 h. The cells were detached from the cell culture plate and re-seeded onto type I collagen (190 μg/ml; Nitta Gelatin NA Inc.) coated cover glasses and allowed to form new adherens junctions at the cell-cell contacts for 24 h. The cells were washed with PBS (pH 7.0) with Ca^2+^ and Mg^2+^ and fixed with 4% formaldehyde and immunostaining was proceeded for further analysis.

### Calcium chelating with EGTA

MSCs were placed onto cover glasses coated with type I collagen (190 μg/ml; Nitta Gelatin NA Inc.). The cells were then incubated in a serum-free condition overnight and treated with TGF-β (1 ng/ml) for 24 h. To disrupt the calcium-dependent adherens junctions, the cells were exposed to EGTA (4 mM, Sigma-Aldrich) for 30 min. Afterward, the cells were fixed with 4% formaldehyde for immunostaining. To recover the junctions affected by EGTA treatment, the culture media containing EGTA was replaced with fresh media, and the cells were allowed to incubate for 60 min and fixed for further analysis.

### Lentivirus transduction

For expression studies, the 3rd generation of lentiviruses was produced in HEK293T cells by co-transfecting pMDLg/pRRE, pRSV/REV, pBaEVRT, and transfer vector (pLL-CMV-ZO-1-Myc or pLL-CMV-ZO-1 ΔSH3-GUK-Myc). The transfection was performed using lipofectamine 3000 reagents (Thermo Fisher Scientific) and the media was replaced with fresh culture media 4 h after the transfection. HEK293T cells were incubated to permit recombinant lentivirus production for 3 days and the supernatant was collected and filtered with a 0.45 μm syringe filter (Millipore, Burlington, MA, USA). To concentrate viral particles to 10 folds, Lenti-X™ Concentrator (Takara) was used followed by the manufacturer’s protocol. MSCs were transduced with recombinant lentivirus supplemented by protamine sulfate (10 μg/ml, Sigma-Aldrich), centrifuged at 700 × *g* for 90 min at 32 °C, and then the culture media were replaced with fresh culture media.

### Plasmid constructs

To prepare the ZO-1 expressing vector (pLL-CMV-ZO-1-Myc), the full-length human ZO-1 tagged with Myc was amplified from pCB6 ZO1 (addgene #30317, Watertown, MA, USA) using the sense primer 5′- tcggatccaggcctcccgccaccatgTCCGCCAGAGCTGCGGCCGC-3′ which is containing complementary sequences flanking the 5′ upstream SmaI cutting point on pLL-CMV-GFP and the pair region of N-terminus of ZO-1. The antisense primer 5′- acctcgaggcatgccccTTACAAGTCCTCTTCAGAAATGAGCTTTTGCTCGGTGGCGACCGGTGGAAAGTGGTCAATAAGGACA-3′ contained complementary sequences flanking the 3′ downstream SmaI cutting point, complementary sequences of coding Myc and 5 amino acids as a linker, and complementary sequence of C-terminus ZO-1. The amplification was conducted using Q5 Hot Start High-Fidelity 2X Master Mix (New England Biolabs, Ipswich, MA, USA) and the PCR product was cloned in pLL-CMV-GFP linearized with SmaI restriction enzyme using In-Fusion® HD Cloning Kit (Takara). pLL-CMV-ZO-1 ΔSH3-GUK-Myc was obtained by removing SH3 and GUK domains (residues 516–779) from pLL-CMV-ZO-1-Myc using site-directed mutagenesis. The mutant was created using Pfu Turbo DNA polymerase (Stratagene) with sense primer 5′-ATCGTCGCATTGTAGAATCAGATGTAGATGGTTGGTATGGTGCGCT-3′ and antisense primer 5′-AGCGCACCATACCAACCATCTACATCTGATTCTACAATGCGACGAT-3′. The PCR cycling conditions used in the site-directed mutagenesis were 15 cycles of denaturation at 95 °C for 15 s, annealing at 55 °C for 15 s and extension at 72 °C for 7 min. Amplified mixtures were treated with DpnI (New England Biolabs) at 37 °C for 1 h.

### Transwell migration assay

MSCs were serum-starved with DMEM/LG supplemented with 2% FBS, 2 mM L-glutamine, and 100 U/ml P/S overnight and the cells were placed into transwell inserts (8 μm pore size; VWR International, Radnor, PA, USA) coated with type I collagen (5 μg/ml; Nitta Gelatin NA Inc.). After 6 h of attachment, the media of the lower chamber was changed to serum-free media containing TGF-β (10 ng/ml) or the conditioned media derived from MDA-MB-231, MCF7, BT474, T47D, SKBR3, PC3, LNCaP, or U87 and incubated for 12 h to induce migration. If necessary, the cells were pretreated with SB505124 (500 nM) for 30 min prior to TGF-β treatment. When the migration of ZO-1- or α-catenin-knockdown MSCs was evaluated, MSCs were transfected with the control siRNA, ZO-1 siRNA, or α-catenin siRNA for 12 h prior to serum starvation and used for further analysis. The inserts were rinsed twice with PBS and fixed with 4% formaldehyde in PBS and stained with DAPI (Thermo Fisher Scientific). The membranes cut off from the insert were mounted on glass slides using Fluoromount-G (SouthernBiotech) and imaged with a Zeiss LSM 700 confocal microscope (Carl Zeiss). The number of cells on either the lower or upper surface of the membrane were counted in 4–5 randomly selected areas per membrane and the percentage of migrated cells was quantified.

### Sphere migration assay

MSCs were suspended at a 1.67 × 10^5^ cells/ml concentration in complete culture media. The 30 μl of the cell suspension was seeded onto the non-adherent lid of the petri dish (SPL Life Sciences, Gyeonggi-do, Republic of Korea) and the lid was turned upside-down and incubated for 3 days to prepare the spheroid of MSCs (~5 × 10^3^ cells/ spheroid). Spheroids were collected with the culture media and seeded onto type I collagen (190 μg/ml; Nitta Gelatin NA Inc.)-coated 24-well plates. They were attached for 12 h and the media was replaced with DMEM/LG supplemented with 2 mM L-glutamine and 100 U/ml P/S for serum starvation and incubated for another 6 h. TGF-β (1 ng/ml) was added into a media to induce MSCs directional migration. After 24 h, the migration area was measured, and the initial volume of the sphere taken at 0 h was removed to calculate the migration index (px) using ImageJ software (NIH).

### Two-well insert migration assay

A two-well culture insert (Ibidi, Gräfelfing, Germany) was attached to a μ-Dish 35 mm, low Grid-500 (Ibidi). MSCs were seeded into the culture insert at a density of 7 × 10^3^ cells/insert. This insert acted as a barrier, ensuring that the cells reached confluence and formed a starting line for chemotactic migration. When ZO-1 knockdown was required, the control siRNA or ZO-1 siRNA was transfected into the MSCs and incubated for 3 days before seeding the cells into the insert. The cells were allowed to adapt overnight, and then the media was replaced with DMEM/LG supplemented with 2 mM L-glutamine and 100 U/ml P/S for serum starvation. After incubating overnight, the insert was gently removed using clean forceps, and the conditioned media from MDA-MB-231 (MDA CM) or the control conditioned media (CON CM) were added to the dish to induce MSCs migration. The migration process was allowed to proceed for 48 h and fixed for immunostaining.

### Three-dimensional (3D) cell migration assay

MSCs transfected with the control siRNA or ZO-1 siRNA for 3 days were prepared and the control siRNA transfected cells were labeled with CellTracker green CMFDA (10 μM). To conduct the migration assay, each group of cells was mixed in a final suspension of 4.5 × 10^5^ cells in 10 μl (in a 1:1 ratio). Then, the suspension was mixed with 50 μl of type 1 A collagen solution (final concentration: 1.5 mg/ml, Nitta Gelatin NA Inc.), 10 μl of 10× reconstitution buffer (2.2 g NaHCO_3_ and 200 mM HEPES in 0.05 N NaOH), 9 μl of 10× Minimum Essential Medium (Thermo Fisher Scientific), and 21 μl of ultrapure distilled water. Subsequently, 3 μl of this collagen mixture was placed onto 12-well plates as a drop and incubated for 20 min at 37 °C to allow solidification. Then, the serum starvation media (DMEM/LG supplemented with 2 mM L-glutamine and 100 U/ml P/S) was added to 12-well plate and incubated for 6 h. The media were then replaced to MDA CM or the conditioned media from MCF7 and MSCs were allowed to migrate. The migration capacity of MSCs transfected with the control siRNA or ZO-1 siRNA was assessed after 36–48 h. To analyze the migration, images were taken using Axio Observer.7 (Carl Zeiss). Cells migrated out from the collagen gel drop were identified as migrated cells and their percentage was measured.

### Statistical analysis

All the quantifications are shown as mean ± standard deviation (SD) and statistical significance was evaluated using unpaired Student’s *t*-test on comparison two groups. Graphic and statistical work was accomplished by Graphpad Prism 9.4 (San Diego, CA, USA) and *p* < 0.05 were considered significant.

### Reporting summary

Further information on research design is available in the Nature Research Reporting Summary linked to this article.

### Supplementary information

supplementary figures and legends

Original Data File

Reporting Summary

## Data Availability

All data generated or analyzed during this study are included in this article and its supplemental data.
