# Supplementary material for: ZO-1 regulates the migration of mesenchymal stem cells in cooperation with α-catenin in response to breast tumor cells
Source: Cell Death Discov. 2024 Jan 11;10:19. doi: 10.1038/s41420-023-01793-4 (PMC10784548; doi:10.1038/s41420-023-01793-4)
Supplement: Supplementary file 1 — supplementary figures and legends [file 41420_2023_1793_MOESM1_ESM.docx]

**ZO-1 regulates the migration of mesenchymal stem cells in cooperation with α-catenin in response to breast tumor cells**

Aran Park^1^, Sanghyuk Choi^1^, Jungbeom Do^2^, Youngjae Kim^2^, Kyung-Sup Kim^3^, Eunjin Koh^3^ and Ki-Sook Park^2,4*^

^1^Graduate School of Biotechnology, Kyung Hee University, Yongin, 17104, Korea

^2^Department of Biomedical Science and Technology, Graduate School, Kyung Hee University, Seoul, 02447, Korea

^3^Department of Biochemistry and Molecular Biology, Institute of Genetic Science, Yonsei University College of Medicine, Seoul 03722, Korea

^4^East-West Medical Research Institute, Kyung Hee University, Seoul, 02447, Korea.

*e-mail: [kisookpark@khu.ac.kr](mailto:kisookpark@khu.ac.kr)

**Supplementary Figures S1 to S5**

**
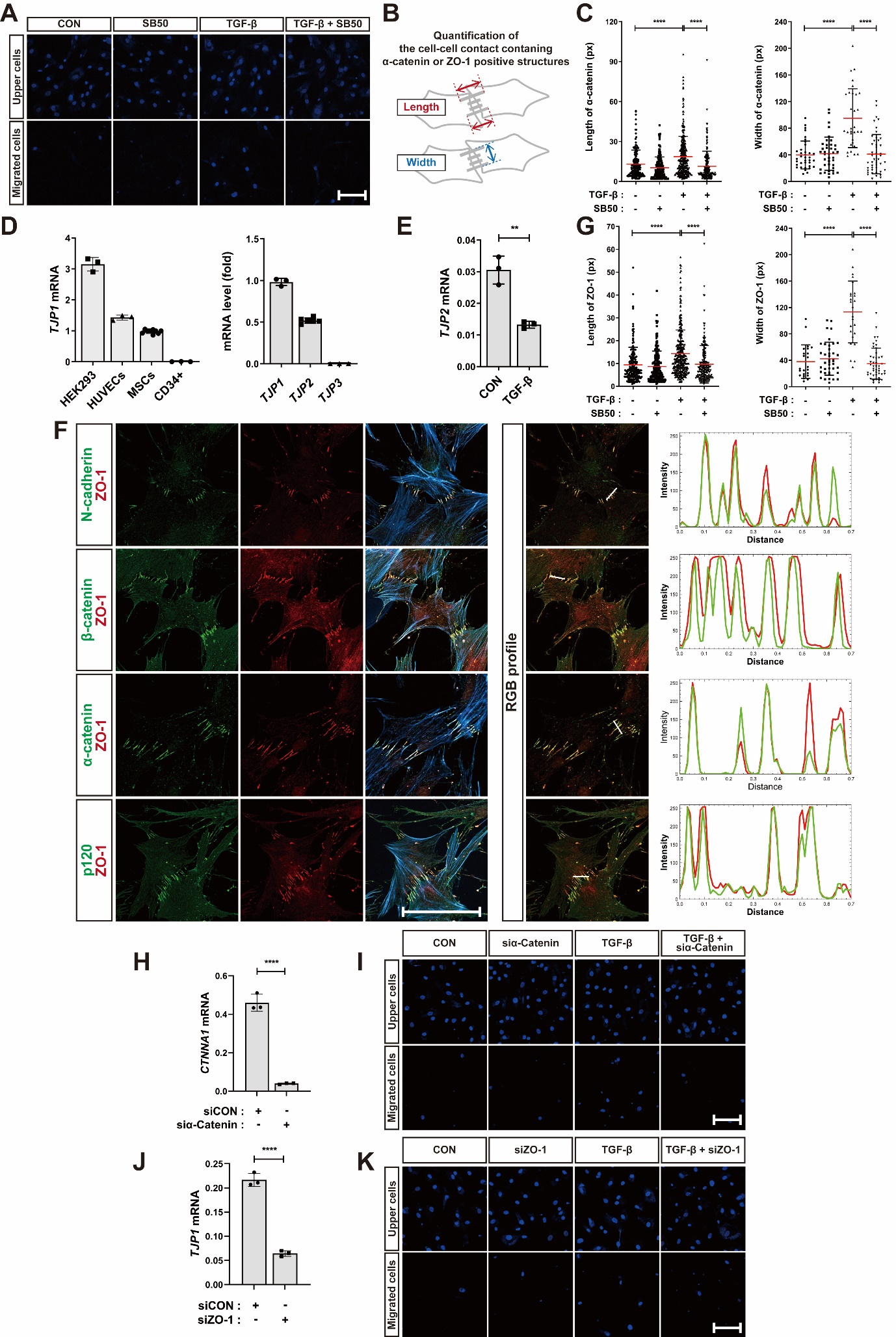
**

**Supplemental Figure S1.** ZO-1 expressed at AJs and mediates MSCs migration in response to TGF-β. **A** Representative image of transwell migration assay results from Figure 1A. DAPI-stained nucleus (blue) of MSCs in both upper (upper cells) and lower side (migrated cells) of the membrane were shown. **B** Scheme for junction quantification of the length of α-catenin-positive or ZO-1-positive structures and the width of the cell-cell contacts containing α-catenin-positive or ZO-1-positive structures on MSCs. **C** The quantification of both the length of α-catenin-positive structures and the width of the cell-cell contacts containing α-catenin-positive AJs on MSCs pretreated with SB505124 (SB50, 500 nM) followed by TGF-β (1 ng/ml) treatment for 24 h (*n*= 2 independent cultures, 23 to 46 regions of cell-cell contacts were analyzed). **D** The expression level of ZO-1 (*TJP1*) mRNA in different types of human cells and the mRNA expression of ZO isoforms (ZO-1, *TJP1*; ZO-2, *TJP2*; ZO-3, *TJP3*) in MSCs. **E** The mRNA expression level of ZO-2 (*TJP2*) in MSCs treated with TGF-β (1 ng/ml) for 24 h. **F** Immunostaining of ZO-1 and AJs-associated proteins (N-cadherin, β-catenin, α-catenin, and p120) in MSCs treated with TGF‐β (1 ng/ml) for 24 h. Actin and nuclei were stained with phalloidin (cyan blue) and DAPI (blue), respectively. RGB profiling was performed using ImageJ in merged channels of ZO-1 and the AJ-associated protein. The analyzed regions are delineated by white lines. **G** The quantification of both the length of ZO-1-positive structures and the width of the cell-cell contacts containing ZO-1-positive AJs on MSCs pretreated with SB505124 (SB50, 500 nM) followed by TGF-β (1 ng/ml) treatment for 24 h (*n=* 2 independent cultures, 23 to 46 cell-cell contact regions were analyzed). **H, I** Knockdown efficiency of α-catenin (**H**) and a representative image (**I**) from Figure 1F. **J, K** Knockdown efficiency of ZO-1 (*TJP1*, **J**) and a representative image (**K**) from Figure 1G. Scale bar, 100 μm. Results are presented as mean ± SD. *P* value measured by unpaired Student’s *t*-test; ***P* < 0.01, *****P* < 0.0001.


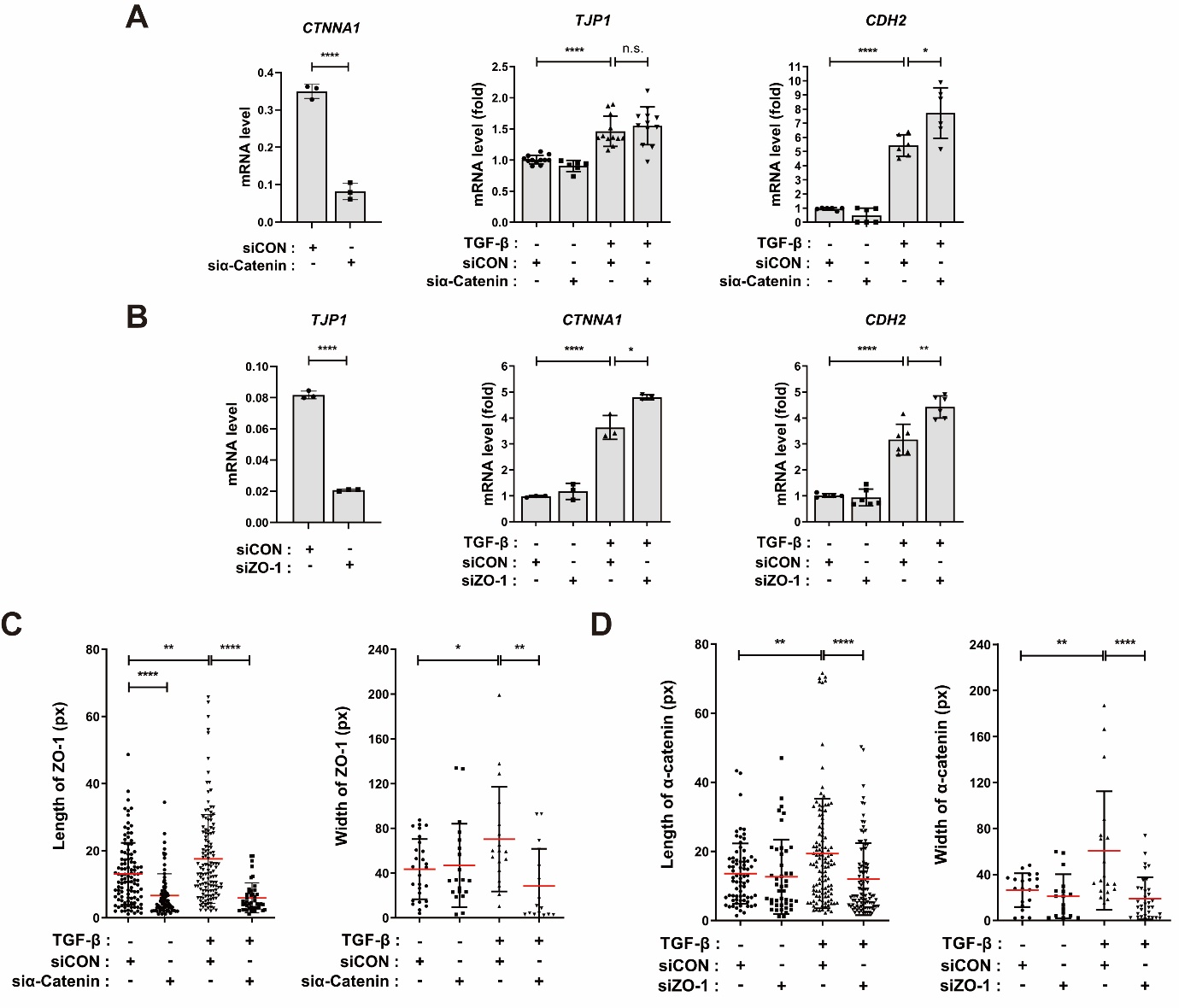


**Supplemental Figure S2.** ZO-1 or α-catenin reciprocally regulates their localization at the cell-cell contacts between MSCs. **A**, **B** The mRNA expression level of α-catenin (*CTNNA1*), ZO-1 (*TJP1*), or N-cadherin (*CDH2*) in α-catenin-knockdown MSCs (**A**) or ZO-1-knockdown MSCs (**B**) (*n*= 4 independent cultures for (**A**) and *n*= 2 independent cultures for (**B**)).. **C** The quantification of both the length of ZO-1-positive structures and the width of the cell-cell contacts containing ZO-1-positive structure on MSCs with α-catenin knockdown (16 to 26 cell-cell contact regions were analyzed). **D** The quantification of both the length of α-catenin-positive structures and the width of the cell-cell contacts containing α-catenin-positive structures on MSCs with ZO-1 knockdown (17 to 34 cell-cell contact regions were analyzed). Results are presented as mean ± SD. *P* value measured by unpaired Student’s *t*-test; **P* < 0.05, ***P* < 0.01, *****P* < 0.0001.

**
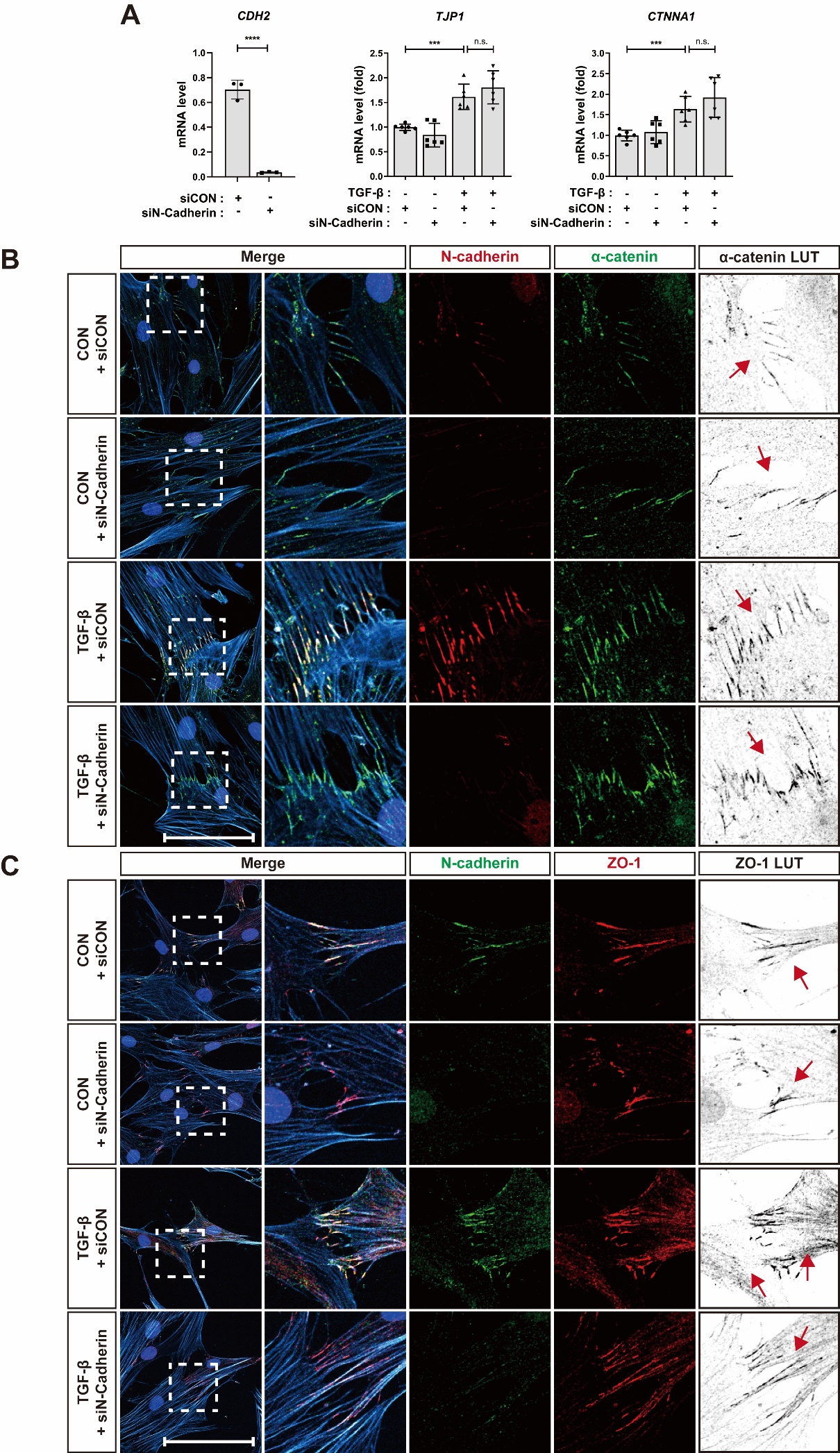
**

**Supplemental Figure S3**. N-cadherin knockdown does not impair the expression of α-catenin and ZO-1 on AJs. **A** The mRNA expression level of N-cadherin (*CDH2*), ZO-1 (*TJP1*), α-catenin (*CTNNA1*) in N-cadherin-knockdown MSCs (*n=* 2 independent cultures). **B, C** Immunostaining of N-cadherin and α-catenin (**B**) or N-cadherin and ZO-1 (**C**) in N-cadherin-knockdown MSCs treated with TGF‐β (1 ng/ml) for 24 h. Actin and nuclei were stained with phalloidin (cyan blue) and DAPI (blue), respectively. To enhance the visibility of α-catenin (**B**) or ZO-1 (**C**), LUT (look up table) inverted images are shown. Red arrows indicate the cell-cell contacts in the regions magnified from white dashed boxes. (*n*= 3 and *n=* 2 independent cultures for α-catenin and ZO-1, respectively). Scale bar, 100 μm. Results are presented as mean ± SD. *P* value measured by unpaired Student’s *t*-test; ****P* < 0.001, *****P* < 0.0001, n.s., not significant.


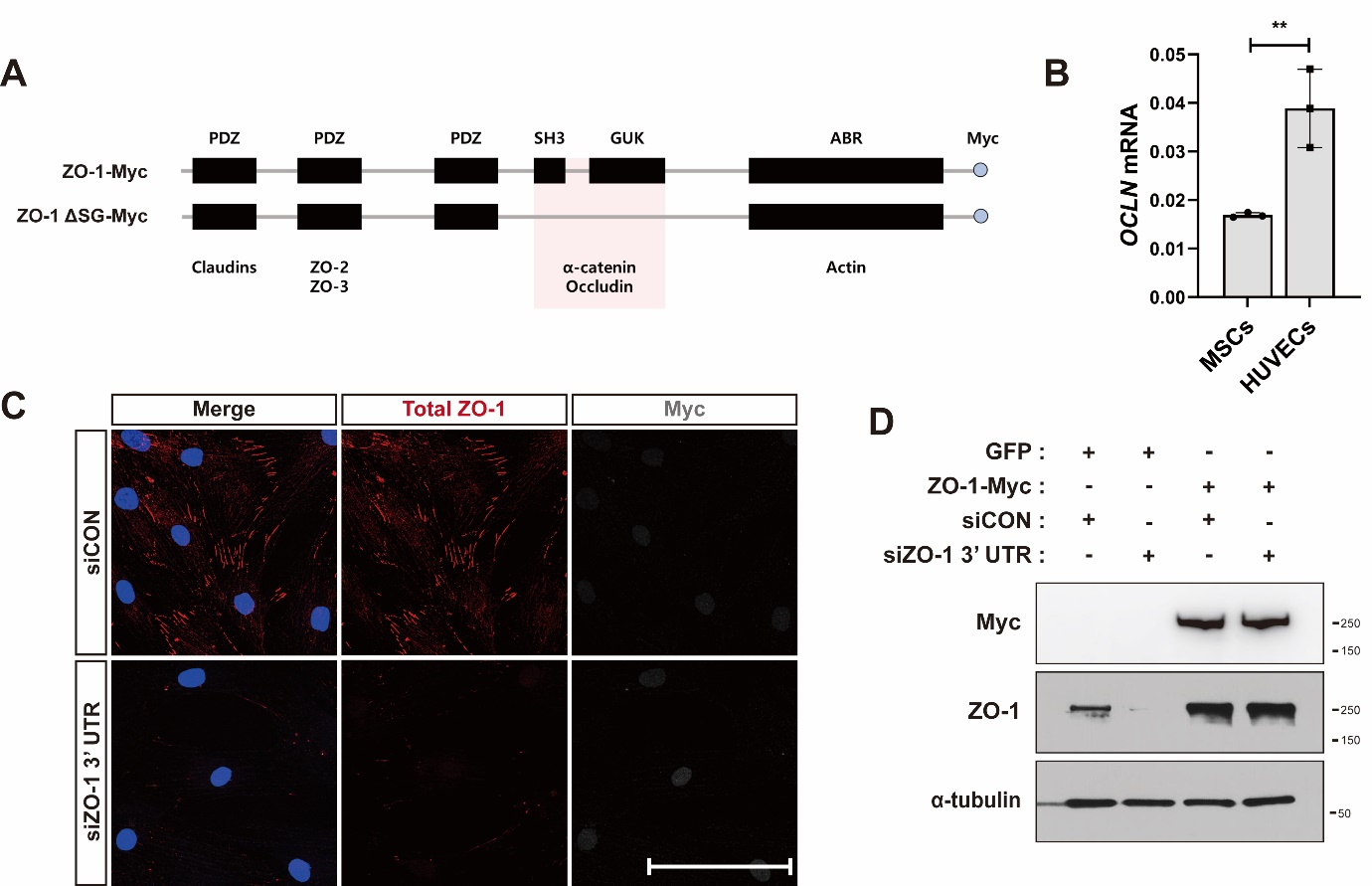


**Supplementary Figure S4.** A mutant ZO-1 which lacks SH3 and GUK domains. **A** Domains of ZO-1. The interaction partners of ZO-1 are delineated in each specific domain. **B** The expression level of occludin (*OCLN*) in MSCs. HUVECs were used as a positive control of occludin expression. *P* value measured by unpaired Student’s *t*-test; ***P* < 0.01. **C** The immunostaining of ZO-1 to confirm endogenous ZO-1 knockdown in MSCs using ZO-1 3’ UTR siRNA (siZO-1 3’UTR). **D** Western blot analysis. Expression of full-length ZO-1 tagged with Myc (ZO-1-Myc) in HEK293 transfected with ZO-1 3’ UTR siRNA (siZO-1 3’UTR) or the control siRNA (siCON).


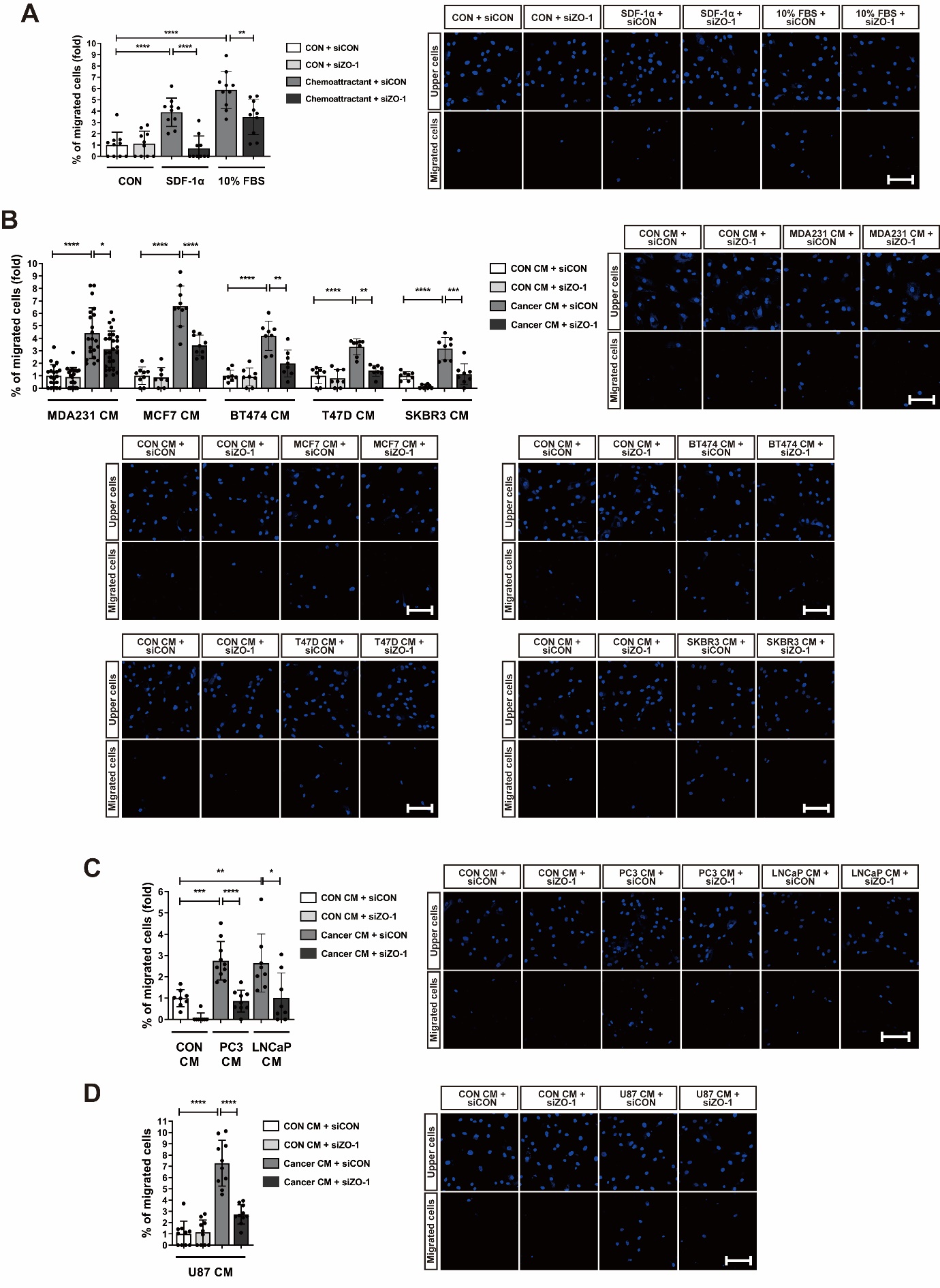


**Supplemental Figure S5.** ZO-1 mediates MSCs migration in response to several chemoattractants and the conditioned media from various cancer cell lines. **A** Transwell migration assay was performed to quantify the migration capacity of MSCs in response to general chemoattractants including SDF-1α (50 ng/ml) or 10% FBS for 12 h (*n*= 2 and *n*= 4 independent cultures in response to SDF-1α and FBS, respectively). **B** Transwell migration assay to quantify MSCs migration in response to conditioned media from breast cancer cell lines (*n*= 4 independent cultures for MDA CM, *n*= 2 for CM of MCF7, BT474, T47D, and SKBR3). **C** Transwell migration assay to quantify MSCs migration in response to conditioned media from prostate cancer cell lines (PC3 or LNCaP) (*n*= 2 independent cultures). **D** Transwell migration assay to quantify MSCs migration in response to conditioned media of U87, a cell line of glioblastoma (*n*= 2 independent cultures). Scale bar, 100 μm. Results are presented as mean ± SD. *P* value measured by unpaired Student’s *t*-test; **P* < 0.05, ***P* < 0.01, ****P* < 0.001, *****P* < 0.0001.
