# Supplementary material for: ZO-1 regulates the migration of mesenchymal stem cells in cooperation with α-catenin in response to breast tumor cells
Source: Cell Death Discov. 2024 Jan 11;10:19. doi: 10.1038/s41420-023-01793-4 (PMC10784548; doi:10.1038/s41420-023-01793-4)

Full and uncropped western blot for Figure 1C
Lanes 1, 2, 3, 4 are on the figure

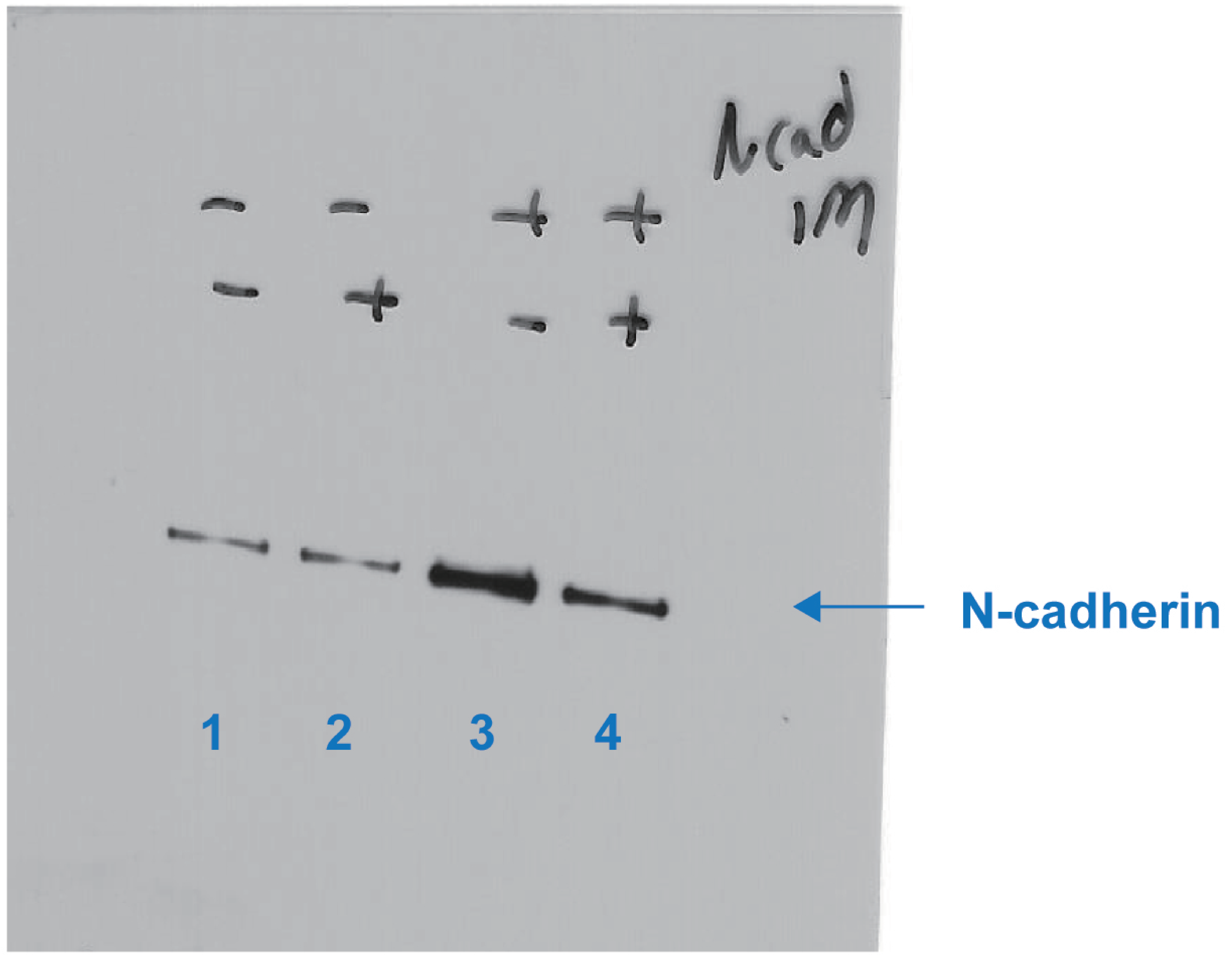

Full and uncropped western blot for Figure 1C
Lanes 1, 2, 3, 4 are on the figure

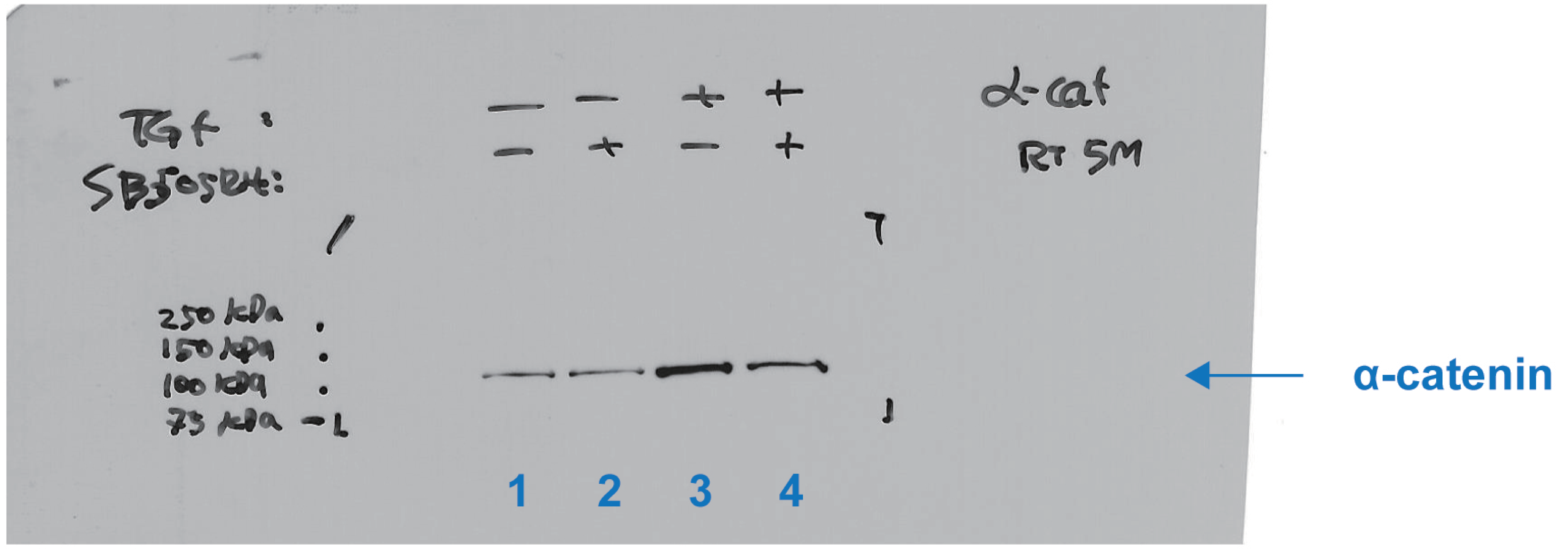

Full and uncropped western blot for Figure 1C
Lanes 1, 2, 3, 4 are on the figure

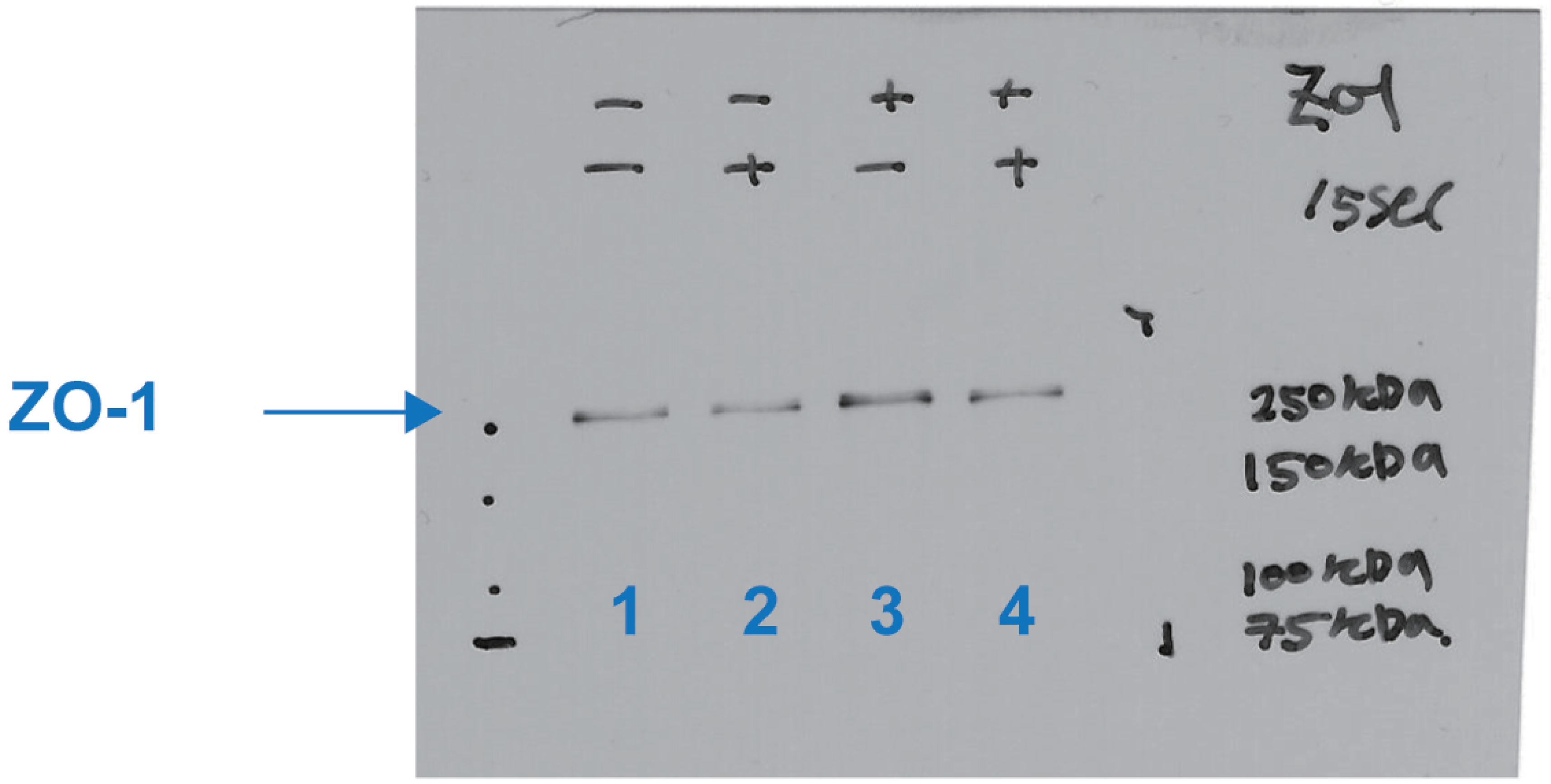

Full and uncropped western blot for Figure 1C
Lanes 1, 2, 3, 4 are on the figure

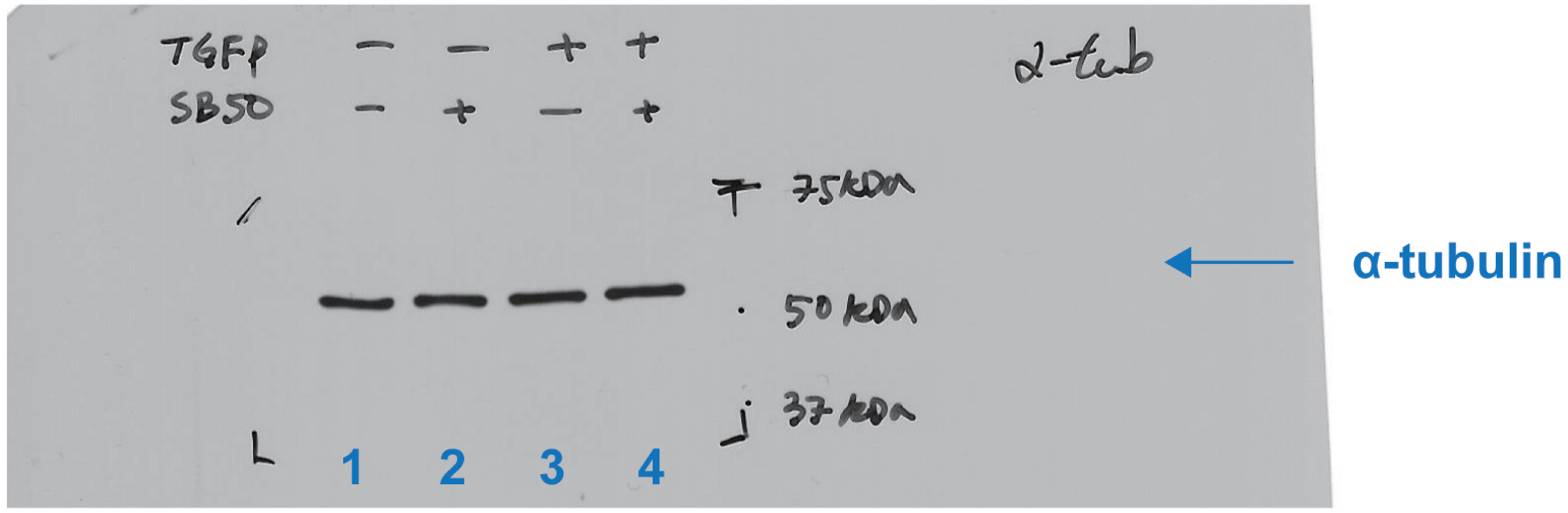

Full and uncropped western blot for Figure 2A
Lanes 1, 2, 3, 4 are on the figure

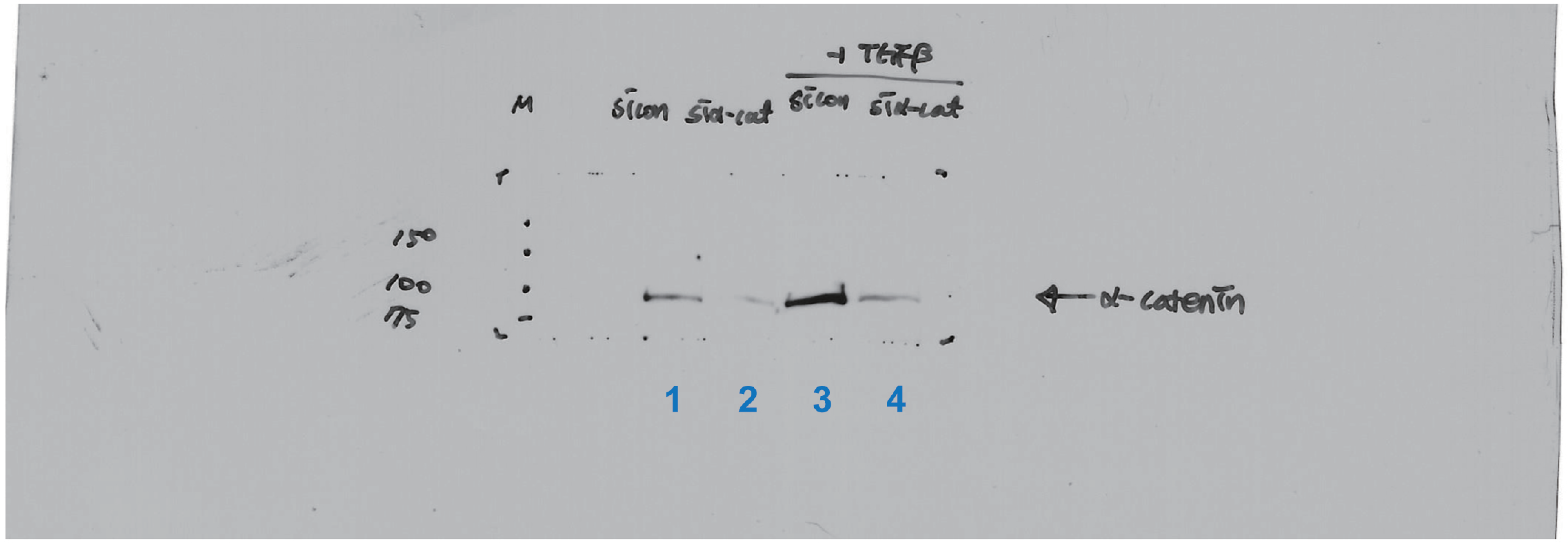

Full and uncropped western blot for Figure 2A
Lanes 1, 2, 3, 4 are on the figure

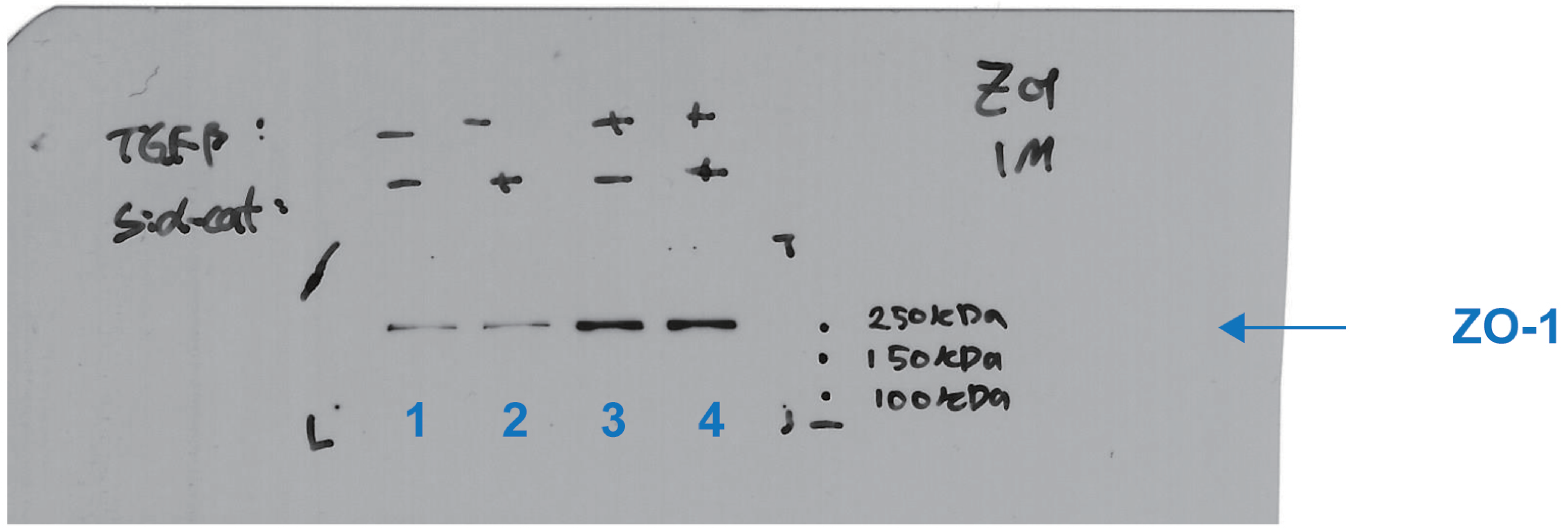

Full and uncropped western blot for Figure 2A
Lanes 1, 2, 3, 4 are on the figure

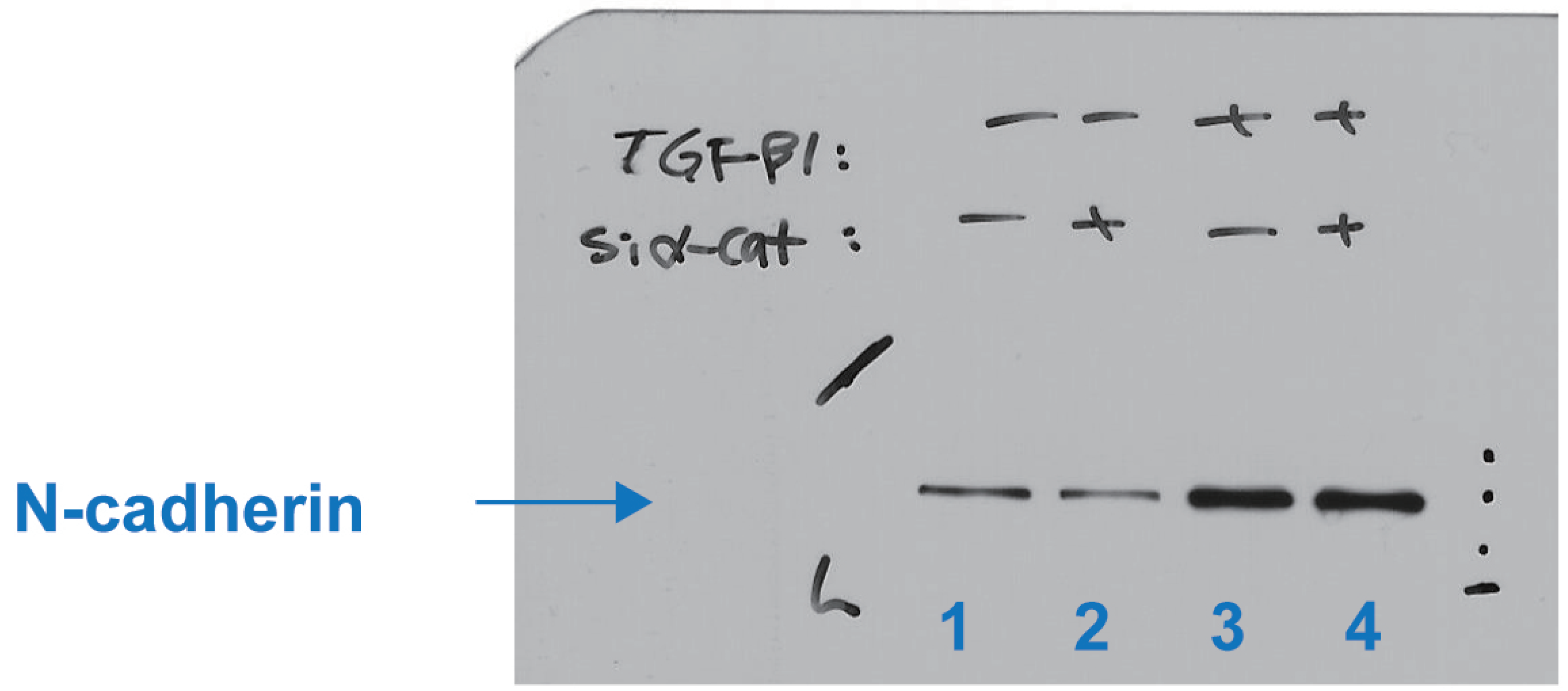

Full and uncropped western blot for Figure 2A
Lanes 1, 2, 3, 4 are on the figure

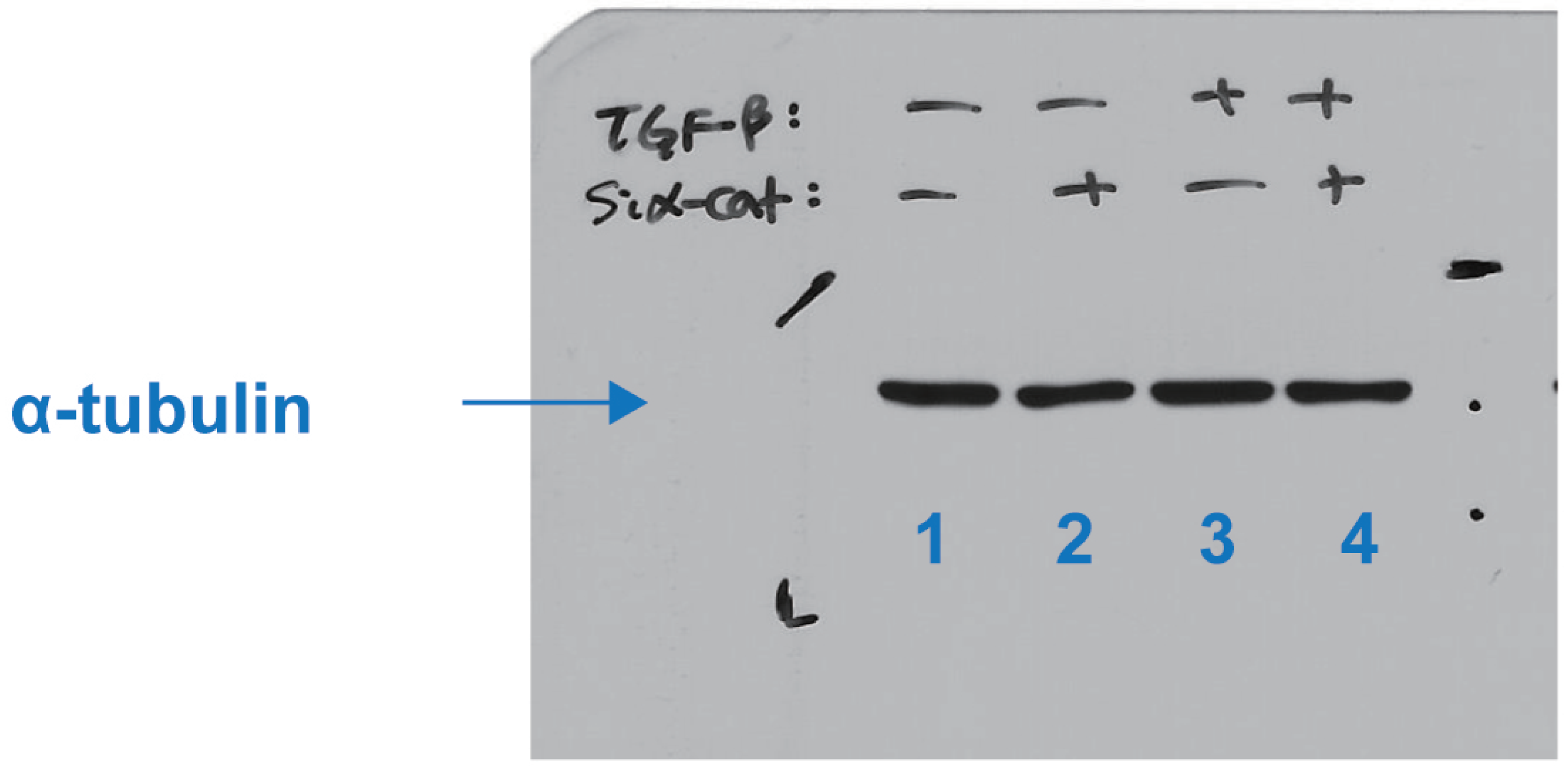

Full and uncropped western blot for Figure 3A
Lanes 1, 2, 3, 4 are on the figure

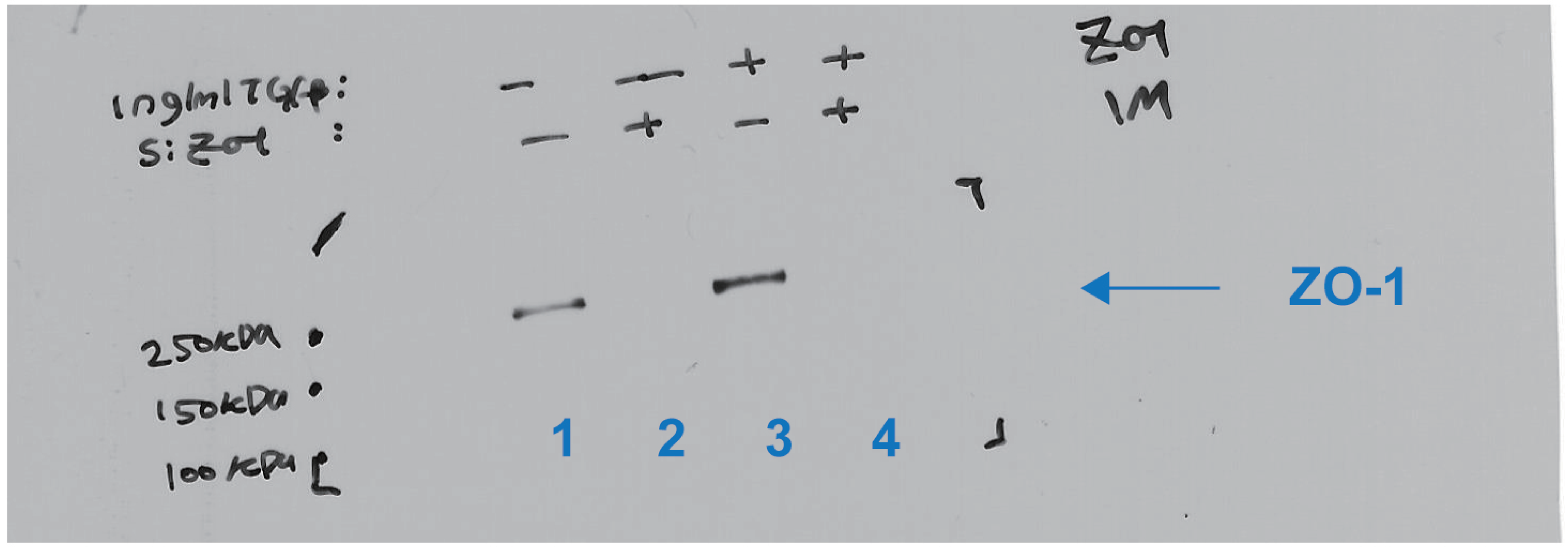

Full and uncropped western blot for Figure 3A
Lanes 1, 2, 3, 4 are on the figure

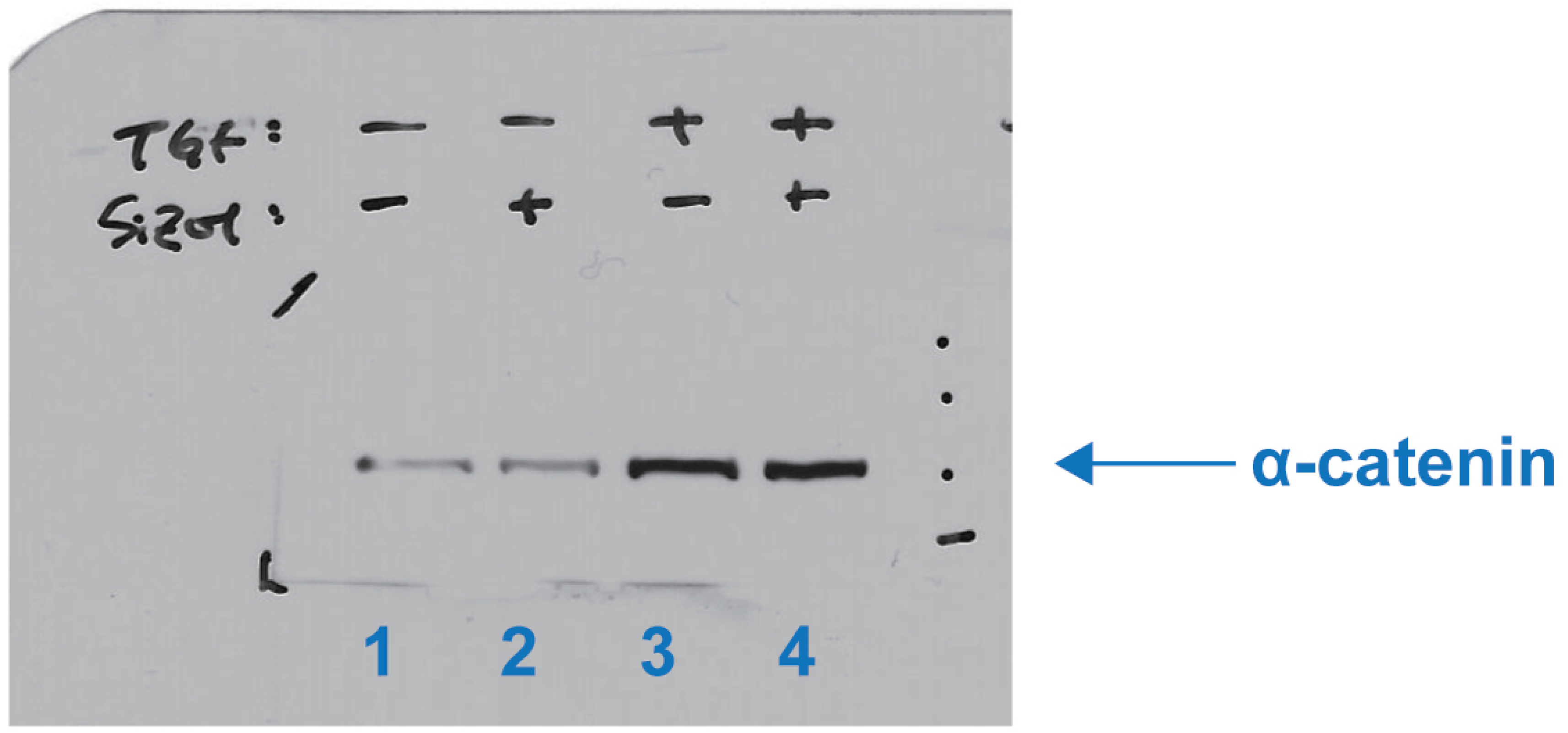

Full and uncropped western blot for Figure 3A
Lanes 1, 2, 3, 4 are on the figure

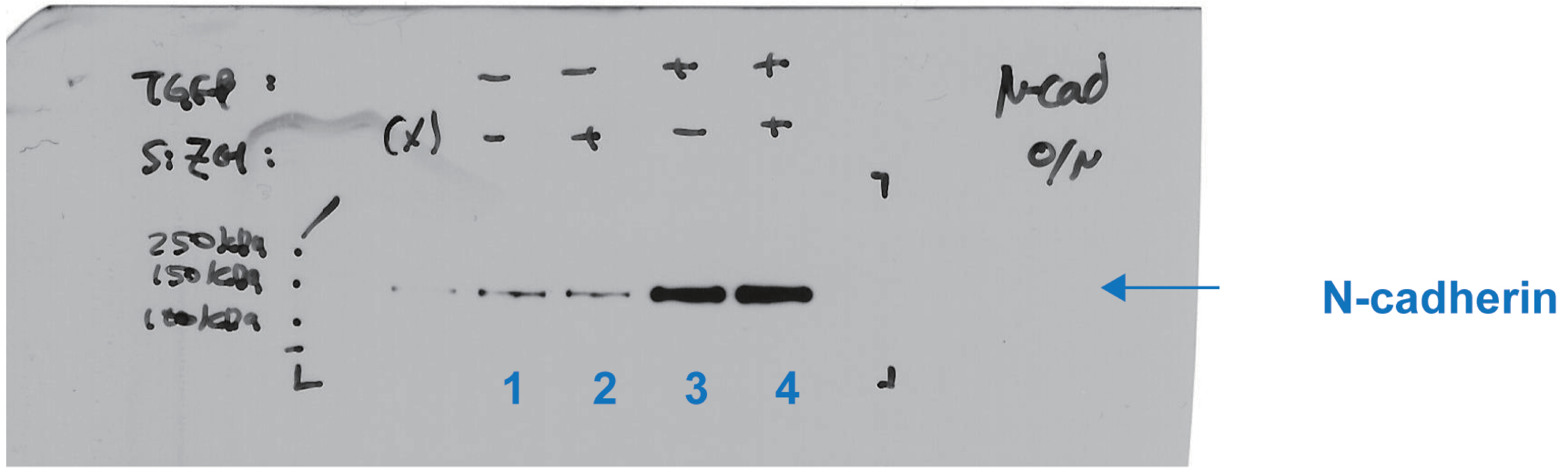

Full and uncropped western blot for Figure 3A
Lanes 1, 2, 3, 4 are on the figure

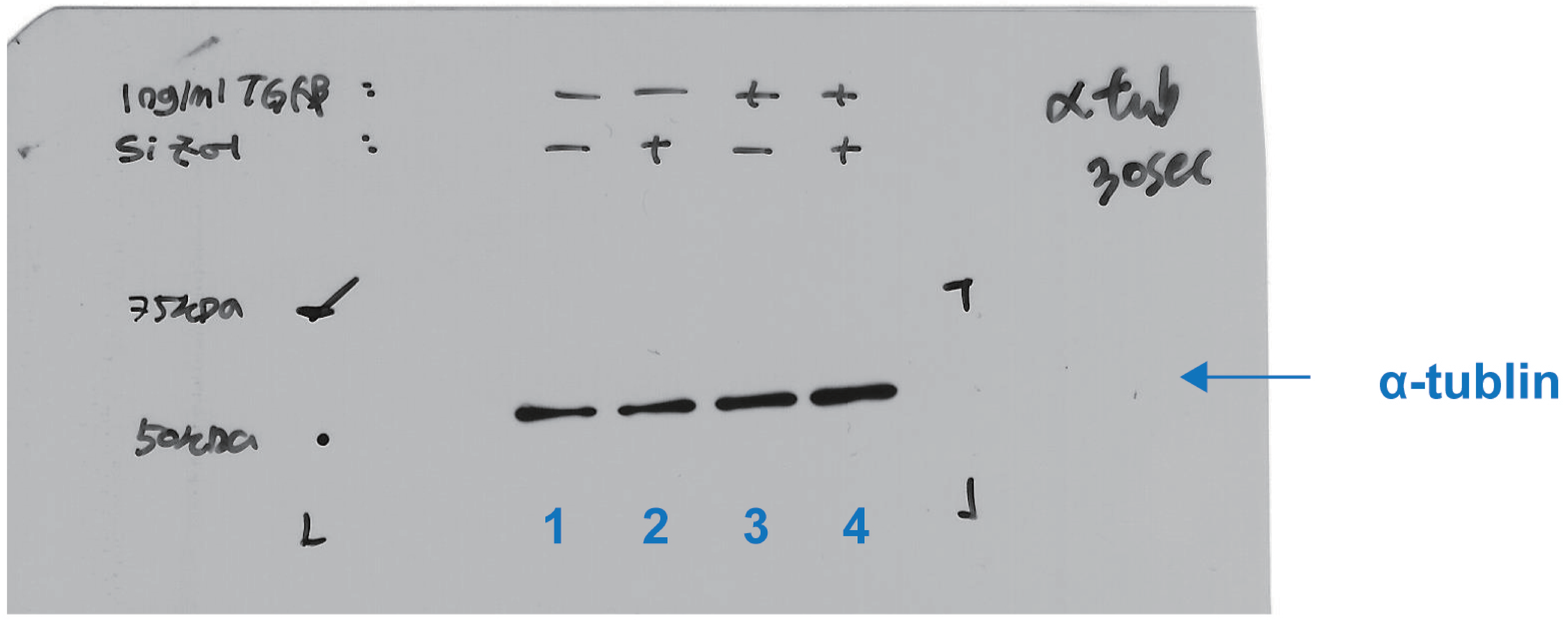

Full and uncropped western blot for Supplemental Figure S4D
Lanes 1, 2, 3, 4 are on the figure

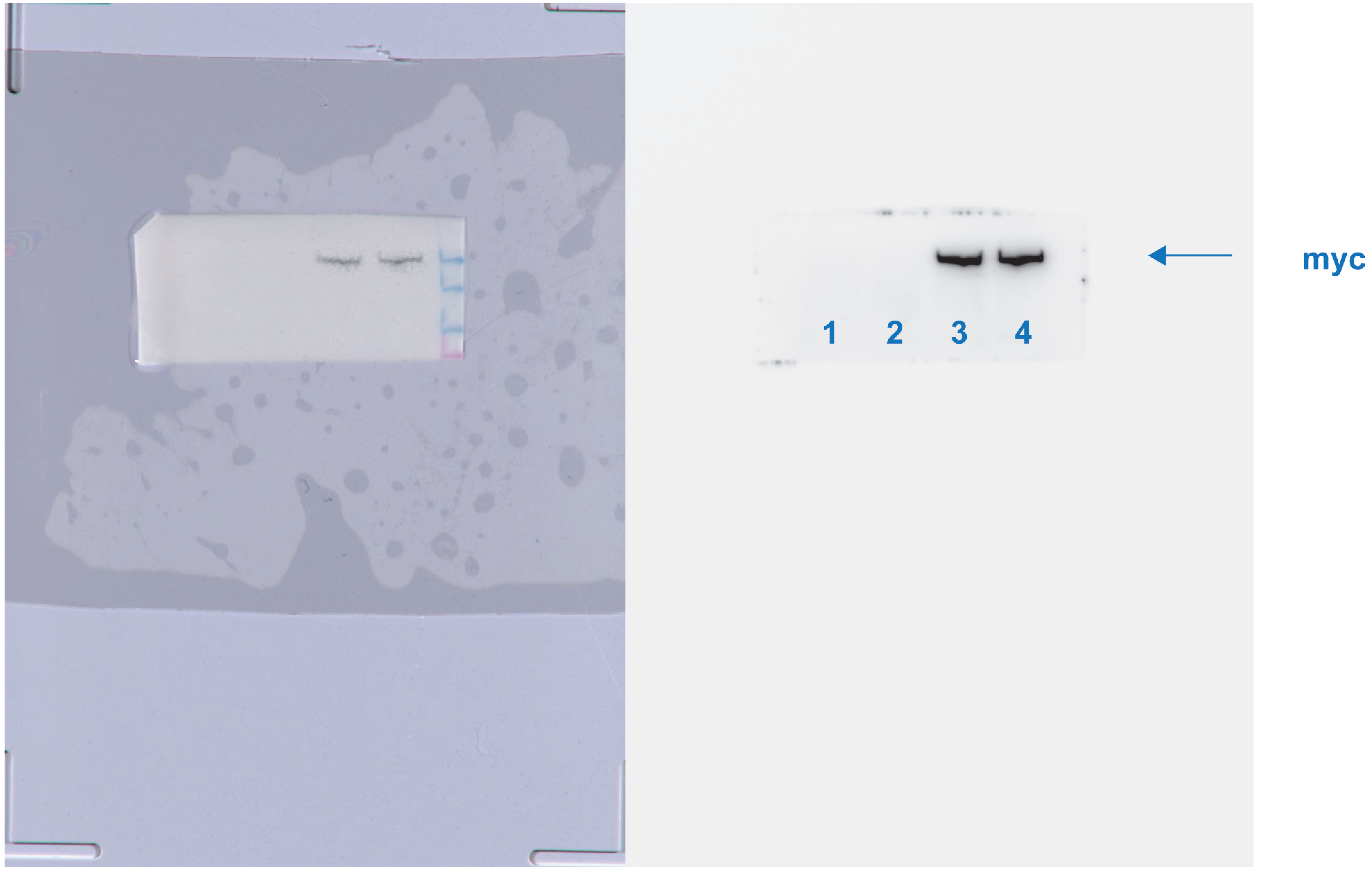

Full and uncropped western blot for Supplemental Figure S4D
Lanes 1, 2, 3, 4 are on the figure

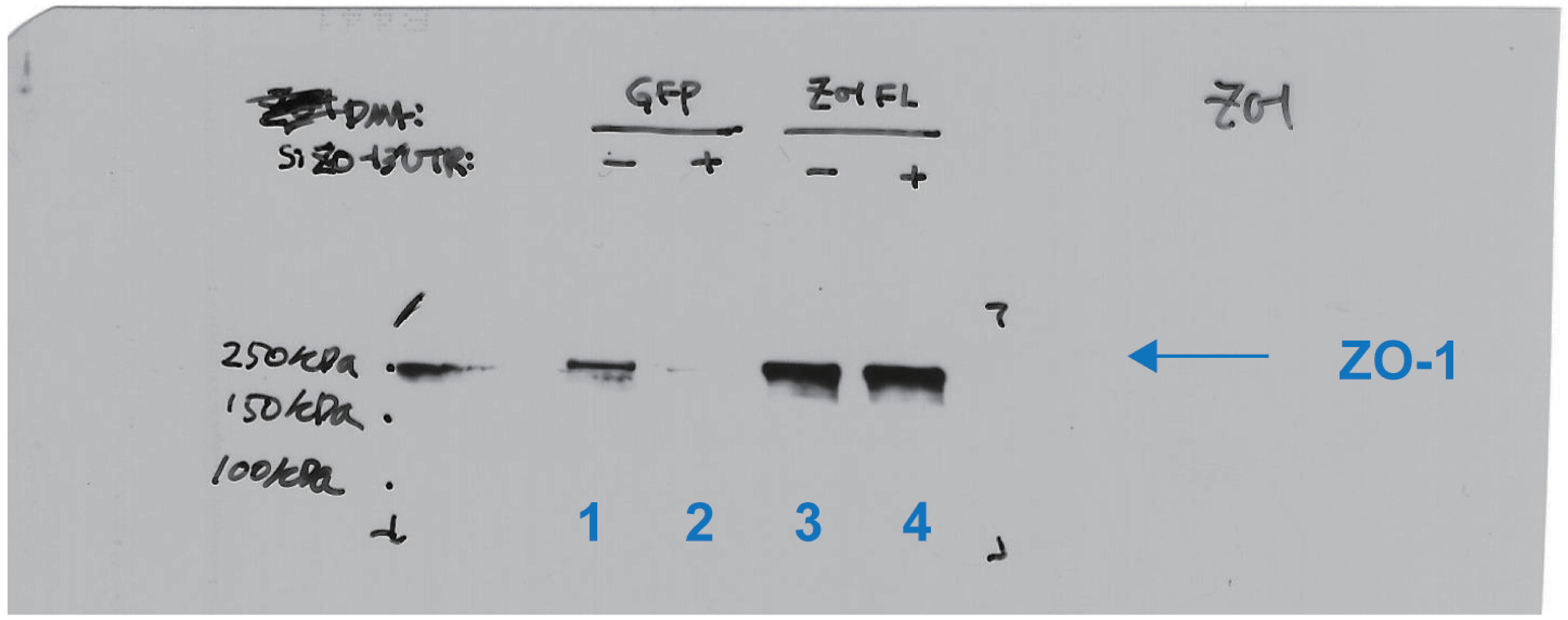

Full and uncropped western blot for Supplemental Figure S4D
Lanes 1, 2, 3, 4 are on the figure

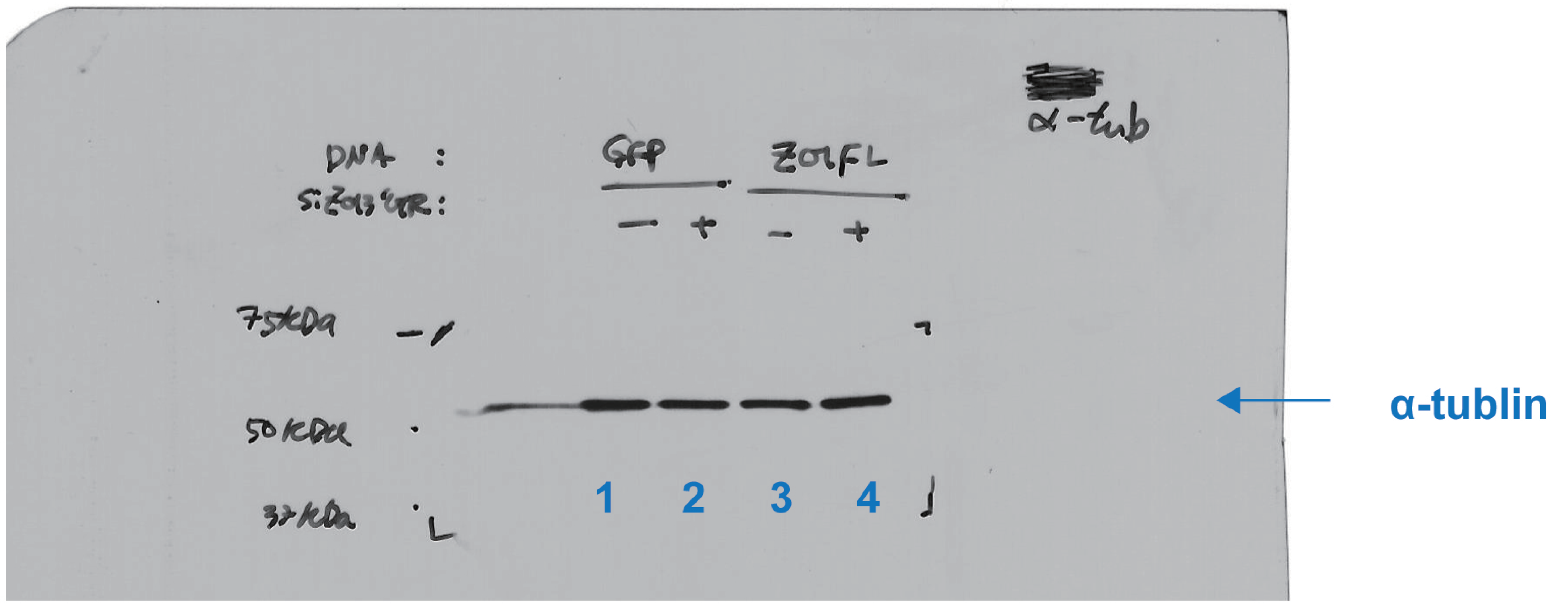

Supplement: Supplementary file 2 — Original Data File [file 41420_2023_1793_MOESM2_ESM.pdf]
